# Selecting reactions and reactants using a switchable rotaxane organocatalyst with two different active sites†
†Electronic supplementary information (ESI) available: Synthetic procedures and characterisation data. See DOI: 10.1039/c4sc03279a


**DOI:** 10.1039/c4sc03279a

**Published:** 2014-11-13

**Authors:** Jack Beswick, Victor Blanco, Guillaume De Bo, David A. Leigh, Urszula Lewandowska, Bartosz Lewandowski, Kenji Mishiro

**Affiliations:** a School of Chemistry , University of Manchester , Oxford Road , Manchester , M13 9PL , UK . Email: david.leigh@manchester.ac.uk ; http://www.catenane.net

## Abstract

The activation mode of a rotaxane-based organocatalyst with both secondary amine and squaramide catalytic units can be switched with acid or base. The macrocycle blocks whichever of the catalytic sites it is positioned over. The switchable rotaxane catalyst generates different products from a mixture of three building blocks according to the location of the macrocyclic ring in the rotaxane.

## Abstract 2

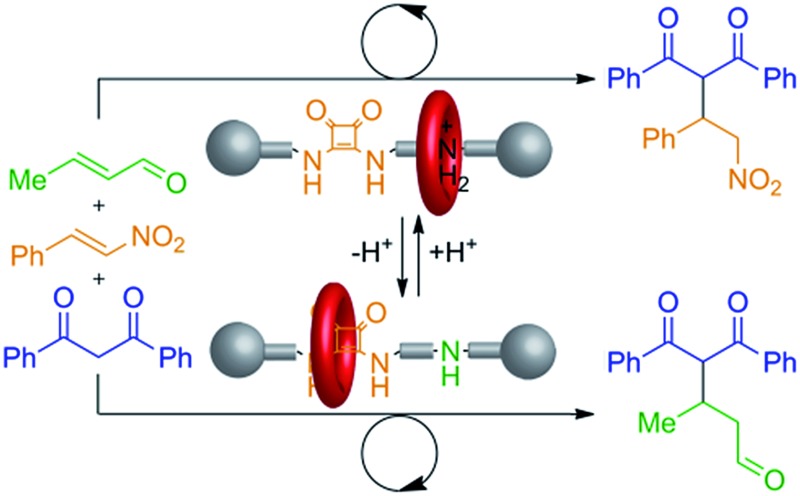
The activation mode of a rotaxane-based organocatalyst with both secondary amine and squaramide catalytic units can be switched with acid or base, affording different products from a mixture of three building blocks.

## Introduction

**Fig. 1:**
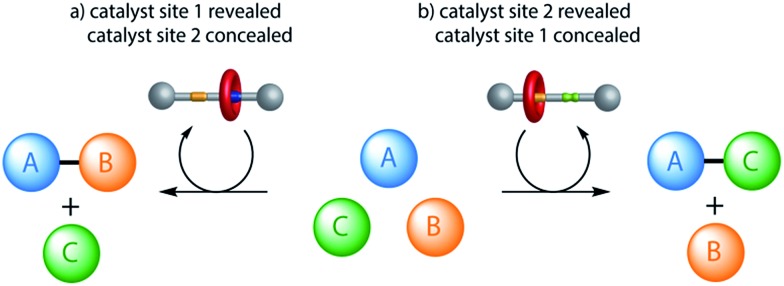
Different products from a mixture of building blocks using a rotaxane catalyst switchable between two different active sites (*e.g.* **1**/**1**-H^+^·CF_3_CO_2_^–^, Fig. 2). Alternative reactions are promoted (involving particular functional groups on different building blocks) according to which active site of the catalyst is revealed (*e.g.* Fig. 4).

Synthetic catalysts have previously been developed where a stimulus can be used to turn the catalytic activity ‘on’ or ‘off’^1,2^ or to change the stereochemical outcome of a reaction.^3^ Here we report on an artificial system that can switch between two different modes of organocatalysis,^4^ each promoting a different chemical transformation. The result is a molecular catalyst that can be used to produce different reaction outcomes from a mixture of building blocks (Fig. 1).

**Fig. 2:**
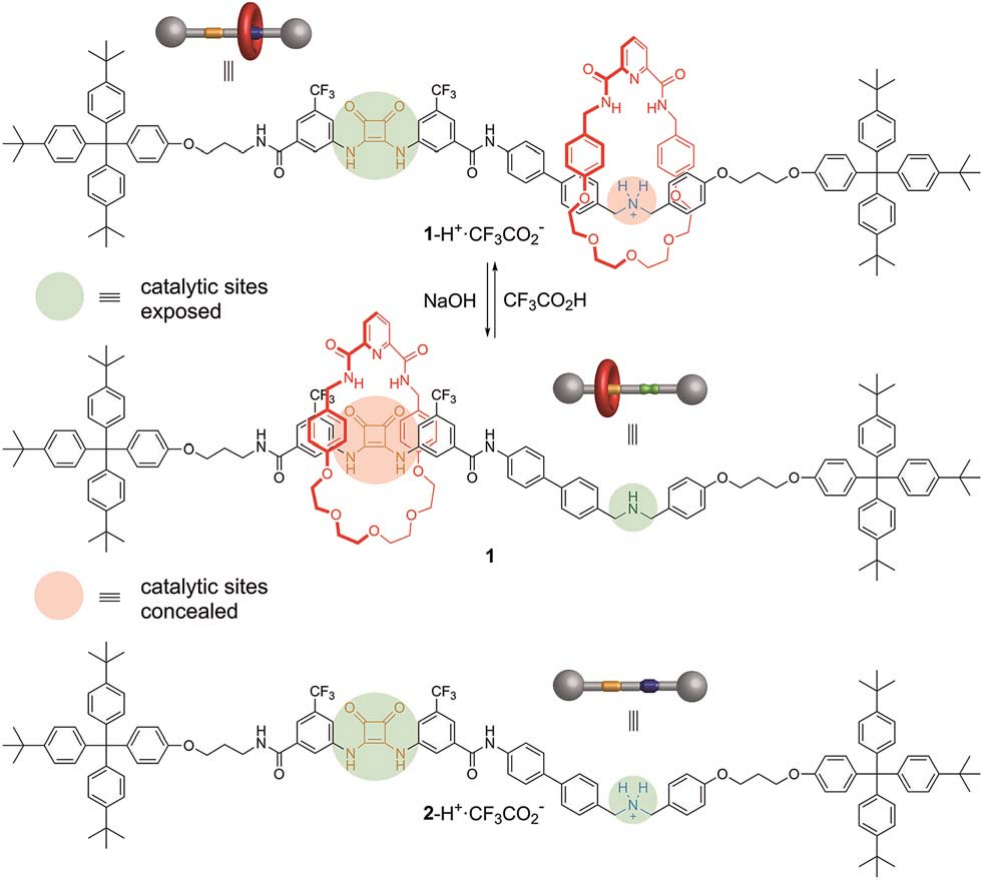
Acid–base control of the position of the macrocycle in rotaxane **1** (iminium catalysis ‘on’; hydrogen bond catalysis ‘off’)/**1**-H^+^·CF_3_CO_2_^–^ (iminium catalysis ‘off’; hydrogen bond catalysis ‘on’) and the structure of the corresponding thread **2**-H^+^·CF_3_CO_2_^–^ (both iminium catalysis and hydrogen bond catalysis ‘on’).

The switchable catalyst employed is a [2]rotaxane in which the position of the macrocycle can be changed^5^ to block one or other of two organocatalytic active sites.^2,6^ The rotaxane (**1**/**1**-H^+^·CF_3_CO_2_^–^, Fig. 2) features a thread bearing dibenzylamine/dibenzylammonium and squaramide units as the catalytic centres. The activities of the organocatalytic sites are based on different activation mechanisms: the secondary amine/ammonium unit is able^2^ to promote iminium^7^ (and potentially enamine^8^ and trienamine^9^) catalysis while squaramide-catalyzed reactions proceed through the activation of electrophiles by hydrogen bonding.^10^ The macrocycle of the rotaxane contains a pyridyl-2,6-dicarboxyamide unit that can bind effectively to the squaramide residue, and a crown ether-like region that has a very high affinity for secondary ammonium ions but not for non-protonated amines.^11^ A rigid spacer was introduced between the two active sites on the thread to prevent folding. Accordingly, when the rotaxane is protonated (**1**-H^+^·CF_3_CO_2_^–^) the macrocycle should preferentially encapsulate the dibenzylammonium group, masking it from being available for catalysis (iminium catalysis ‘off’) while leaving the squaramide site accessible (hydrogen bond catalysis ‘on’). In the neutral form of the rotaxane (**1**) the squaramide should be the preferred binding site for the macrocycle, concealing it and making it unavailable for catalysis (hydrogen bond catalysis ‘off’) whilst leaving the secondary amine exposed (iminium catalysis ‘on’).^12^

## Results and discussion

**Fig. 3:**
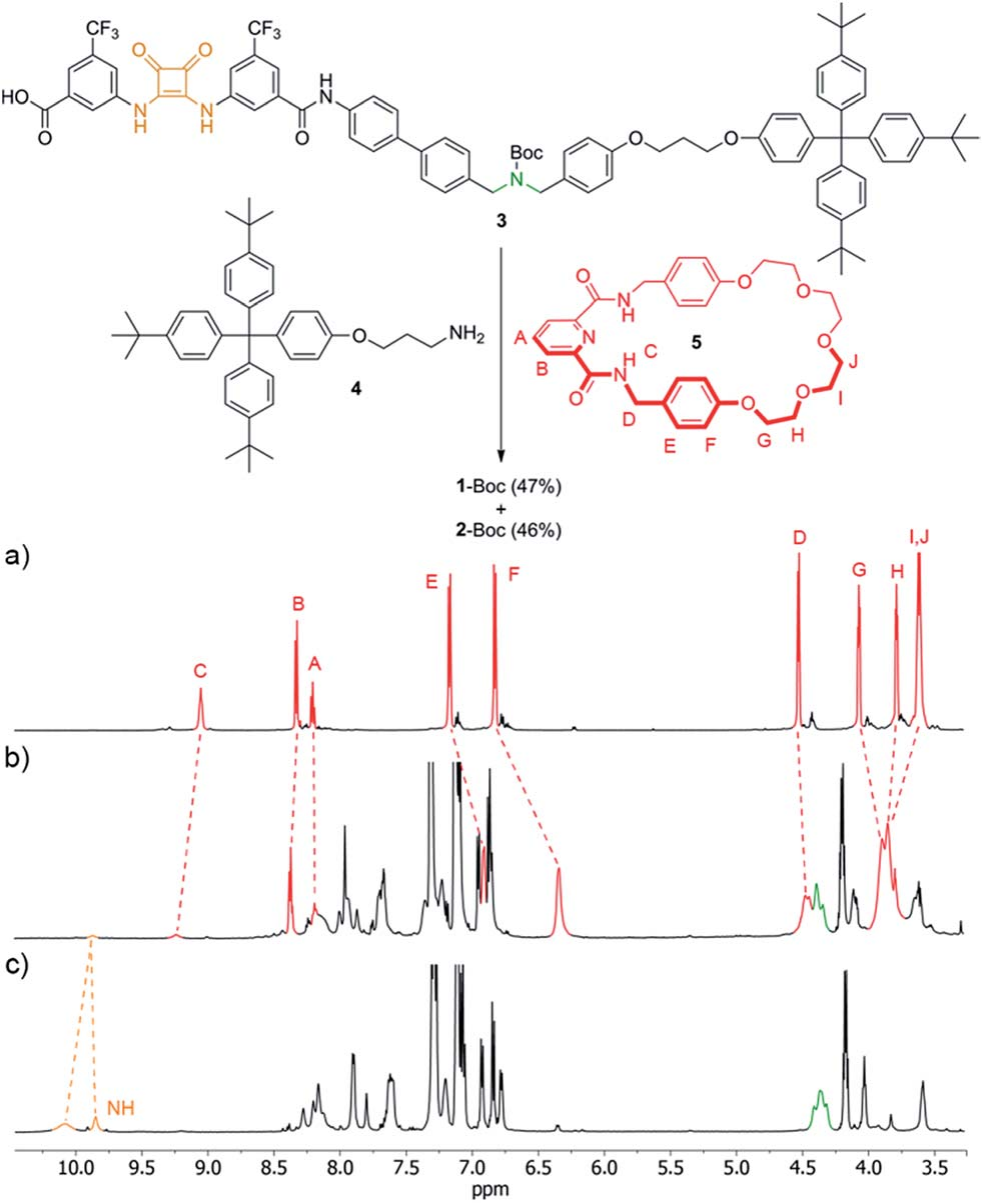
Hydrogen bond mediated assembly of [2]rotaxane **1**-Boc and thread **2**-Boc. Reagents and conditions: PyBroP, iPr_2_NEt, CH_2_Cl_2_ : THF : CH_3_CN (60 : 35 : 5), RT, 20 h. ^1^H NMR spectra (600 MHz, *d*_6_-acetone, 293 K): (a) macrocycle **5**; (b) [2]rotaxane **1**-Boc; (c) thread **2**-Boc.

The synthesis of **1** utilized the intended pyridinedicarboxamide-squaramide recognition motif to promote the threading of a suitable squaramide derivative, **3**, through the cavity of macrocycle **5**, covalently capturing the interlocked structure through amide bond formation with bulky ‘stopper’ **4** (Fig. 3, see ESI† for details). [2]Rotaxane **1**-Boc was isolated in 47% yield along with the non-interlocked thread (**2**-Boc, 46%).

The ^1^H NMR spectra (Fig. 3a–c) of the macrocycle (**5**), thread (**2**-Boc) and rotaxane (**1**-Boc) confirms the threaded architecture of **1**-Boc with the macrocycle residing around the squaramide unit. The downfield shift of the H_C_ amide protons in the rotaxane compared to the parent macrocycle (Δ*δ*H_C_ = 0.19 ppm) and the shifts of the protons on the central region of the polyether chain (Δ*δ*H_H_ = 0.08 ppm; Δ*δ*H_I,J_ = 0.25 ppm) indicate hydrogen bonding between the macrocycle and both sides, hydrogen bond donors and acceptors, of the thread squaramide unit. Protons of the phenyl rings of the macrocycle are shifted upfield in the rotaxane (Δ*δ*H_E_ = –0.26 ppm; Δ*δ*H_F_ = –0.48 ppm) due to shielding by the ring currents of the squaramide ring and aryl substituents.

Deprotection of the dibenzylamine moiety using trifluoroacetic acid afforded rotaxane **1**-H^+^·CF_3_CO_2_^–^ (see ESI† for details). A solution of **1**-H^+^·CF_3_CO_2_^–^ in CH_2_Cl_2_ was washed with NaOH_(aq)_ (2 M) to produce **1**, ^1^H NMR spectroscopy confirming the change of position of the macrocycle (see ESI†). Addition of CF_3_CO_2_H (1.4 equiv.) to **1** in CH_2_Cl_2_ smoothly regenerated **1**-H^+^·CF_3_CO_2_^–^ (see ESI†).

We investigated the ability of the rotaxane and the thread to perform organocatalytic reactions in both their protonated (**1**-H^+^·CF_3_CO_2_^–^; **2**-H^+^·CF_3_CO_2_^–^) and unprotonated (**1**) states. Secondary amines can promote the Michael addition of 1,3-dicarbonyl nucleophiles to α,β-unsaturated aldehydes *via* iminium catalysis.^13^ When using a nitroalkene instead of the unsaturated aldehyde a similar Michael addition can occur if the electrophile is activated by hydrogen bond catalysts such as (thio)urea or squaramide derivatives.^14^ Accordingly, we reasoned that the rotaxane might be able to catalyse the Michael addition of 1,3-diphenylpropane-1,3-dione (**6**) selectively to either crotonaldehyde (**7**) or *trans*-β-nitrostyrene (**8**) according to which type of organocatalytic group was exposed on the thread.

**Fig. 4:**
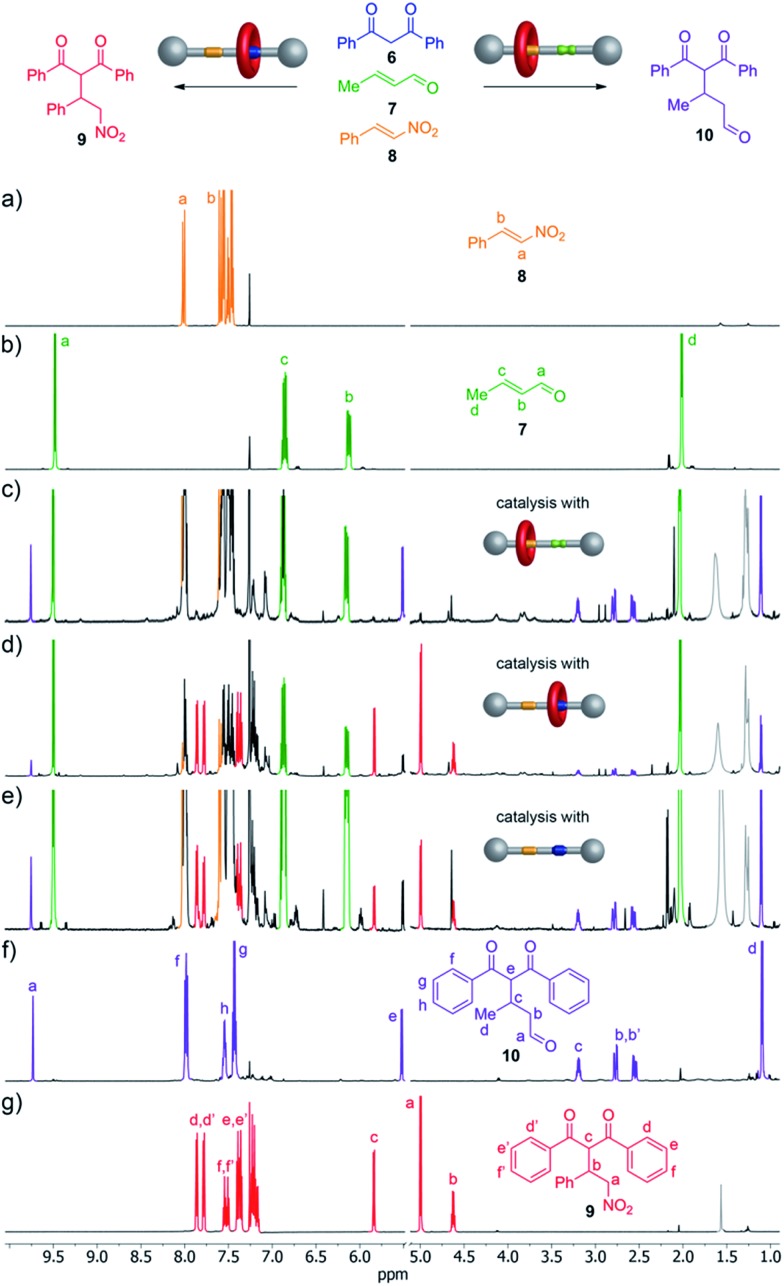
The Michael addition of **6** to crotonaldehyde (**7**) or *trans*-β-nitrostyrene (**8**) using rotaxanes **1**, **1**-H^+^·CF_3_CO_2_^–^ or thread **2**-H^+^·CF_3_CO_2_^–^ as catalysts. Conditions: 5 mol% catalyst, 10 mol% NaOAc, 0.5 M **6** (1 equiv.), **7** (2 equiv), **8** (1 equiv.), RT, 18 h (**1**-H^+^·CF_3_CO_2_^–^) or 72 h (**1** or **2**-H^+^·CF_3_CO_2_^–^). ^1^H NMR spectra (600 MHz, CDCl_3_, 293 K): (a) *trans*-β-nitrostyrene (**8**); (b) crotonaldehyde (**7**); (c) reaction mixture of **6**, **7** and **8** after 72 h in the presence of **1**; (d) reaction mixture of **6**, **7** and **8** after 18 h in the presence of **1**-H^+^·CF_3_CO_2_^–^; (e) reaction mixture of **6**, **7** and **8** after 72 h in the presence of **2**-H^+^·CF_3_CO_2_^–^; (f) **10**; (g) **9**.

A mixture of **6** (0.5 M), **7** and **8** in a 1 : 2 : 1 ratio, 10 mol% NaOAc^15^ and 5 mol% of the potential catalyst (**1**, **1**-H^+^·CF_3_CO_2_^–^ or **2**-H^+^·CF_3_CO_2_^–^) was stirred in CH_2_Cl_2_ at room temperature (Fig. 4, top). Rotaxane **1** (secondary amine exposed) catalyzed the Michael addition of **6** to crotonaldehyde (**7**) to give **10** (40% conversion after 72 h) with high selectivity (only a trace of **9**, the addition product to *trans*-β-nitrostyrene, present in the reaction mixture as evidenced by ^1^H NMR spectroscopy, Fig. 4c). Use of the protonated form of the rotaxane, **1**-H^+^·CF_3_CO_2_^–^, (squaramide exposed) resulted in the formation of **9** with a conversion of 75% after 18 h with only a few percent of **10** present in the reaction mixture (Fig. 4d).

In contrast to the selectivity found with both forms of the rotaxane catalyst, when the thread **2**-H^+^·CF_3_CO_2_^–^ was employed as the catalyst (both organocatalytic sites exposed) **9** and **10** were formed in a close-to-1 : 1 ratio (15% conversion after 72 h, Fig. 4e).

## Conclusions

A rotaxane with two different organocatalytic sites, a squaramide unit and a dibenzylamine group, separated by a rigid spacer, has been demonstrated to promote Michael addition reactions through either iminium ion or hydrogen-bond-activated catalysis. The system can be switched between the two activation modes through acid–base-mediated control of the position of the rotaxane macrocycle to conceal one site on the thread and reveal the other. The switchable organocatalyst was used to promote the Michael addition of 1,3-diphenylpropan-1,3-dione (**6**) to either crotonaldehyde (**7**) or *trans*-β-nitrostyrene (**8**) according to the catalyst state, with modest conversions (40–75%) and good selectivity in both modes.

The ability to select which components of a mixture react together, affording different product outcomes from a common set of building blocks, is a promising use of artificial molecular machines in chemical synthesis.^16^
