# Supplementary material for: Selecting reactions and reactants using a switchable rotaxane organocatalyst with two different active sites
Source: Chem Sci. 2014 Nov 13;6(1):140–3. doi: 10.1039/c4sc03279a (PMC5424444; doi:10.1039/c4sc03279a)
Supplement: Supplementary file 1 [file SC-006-C4SC03279A-s001.pdf]

Electronic Supplementary Material (ESI) for Chemical Science.
This journal is © The Royal Society of Chemistry 2014

Supporting Information

Selecting Reactions and Reactants using a Switchable Rotaxane Organocatalyst with Two Different Active Sites

Jack Beswick,[a] Victor Blanco,[a] Guillaume De Bo,[a] David A. Leigh,[a]* Urszula Lewandowska,[a] Bartosz Lewandowski,[a] and Kenji Mishiro[a]

School of Chemistry, University of Manchester, Oxford Road, Manchester, M13 9PL (UK)

* E-mail: david.leigh@manchester.ac.uk
Homepage: http://www.catenane.net

Table of Contents

1. Experimental Section..S3

1.1. General Methods S3

1.2. Synthesis Overview S4

1.3. Synthetic Procedures and Characterization Details...S7

1.4. ^{1}H NMR spectra for selected compounds S18

2. Additional Supporting Figures S22

3. References and notes S23

1. Experimental Section

1.1. General Methods

Unless stated otherwise, all reagents and solvents were purchased from Aldrich Chemicals and used without further purification. 4-[*tris*-(4-*tert*-butylphenyl)methyl]phenol (**S8**)[1] and compounds **S3**,[2] **5**[3] and **10**[4] were prepared according to literature procedures. Dry THF was obtained by passing the solvent (HPLC grade) through an activated alumina column on a Phoenix SDS solvent drying system (JC Meyer Solvent Systems, CA, USA). Dry MeOH and dry DMF were purchased from Sigma-Aldrich. Peptide grade DMF was purchased from Applied Biosystems. Column chromatography was carried out using Aldrich Si 60 (particle size 40-63 μm) as the stationary phase, and TLC was performed on precoated silica gel plates (0.25 mm thick, 60 F_{254}, Merck, Germany) and observed under UV light. NMR spectra were recorded on a Bruker Avance III 400 or a Bruker Avance III (equipped with a cryoprobe) instrument with an Oxford AS600 magnet. Chemical shifts are reported in parts per million (ppm) from high to low frequency and referenced to the residual solvent resonance. Coupling constants (*J*) are reported in hertz (Hz). Standard abbreviations indicating multiplicity were used as follows: s = singlet, d = doublet, t = triplet, q = quartet, quin = quintuplet, m = multiplet, br = broad. ^{1}H assignments were made using 2D NMR methods (COSY, HSQC, HMBC). Melting points (M. p.) were determined using a Büchi M-565 apparatus and are reported uncorrected. Low resolution ESI mass spectrometry was performed with a Thermo Scientific LCQ Fleet mass spectrometer. High resolution ESI (electrospray ionization) mass spectrometry was carried out by the mass spectrometry services at the University of Manchester.

1.2. Synthesis Overview

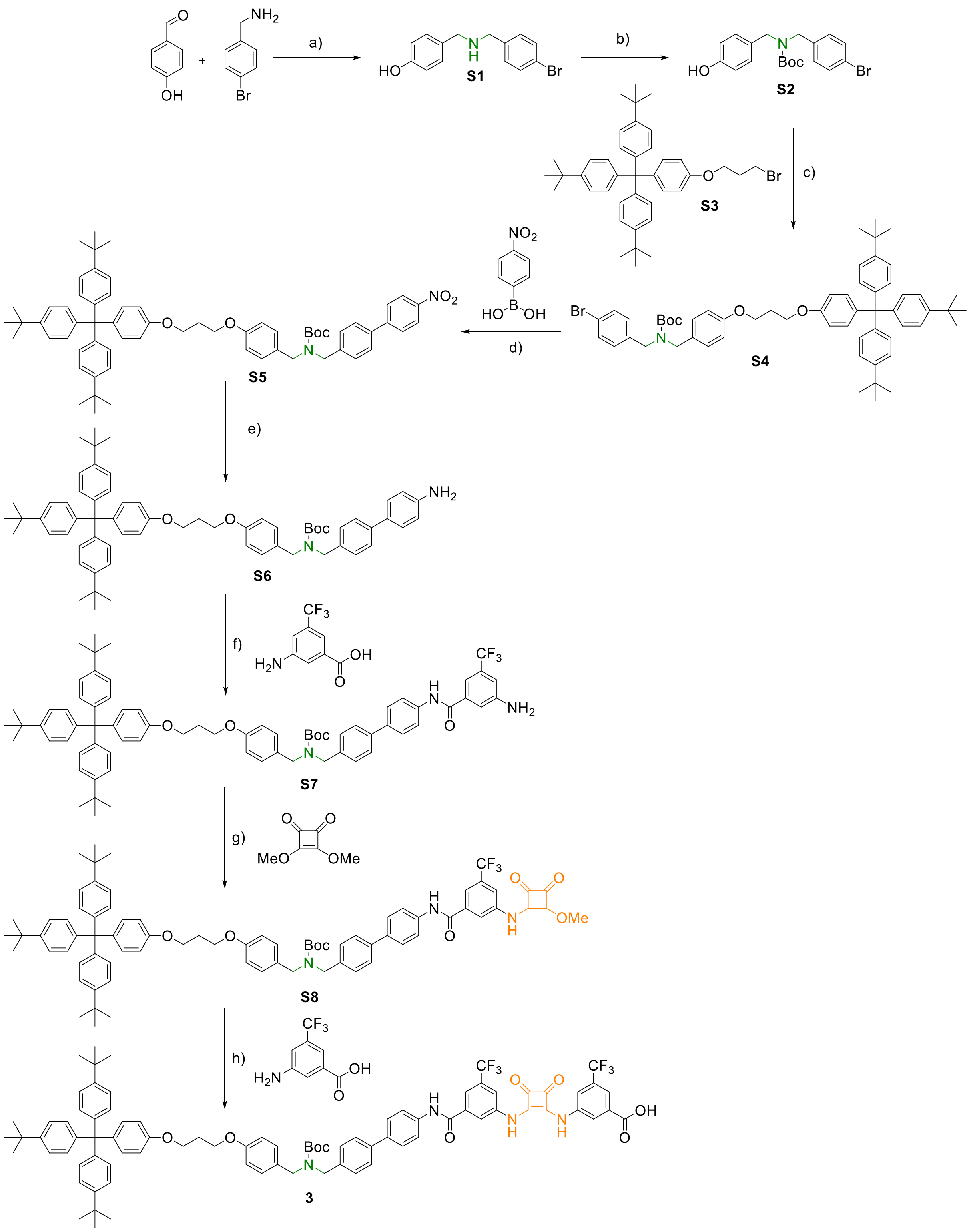

Scheme S1. Synthesis of compound **3**. Reagents and conditions: a) i) MeOH, RT, O/N; ii) $NaBH_4$, THF/MeOH, RT, O/N, 89%. b) Boc_2O, Et_3N, MeOH, RT, O/N, 84%. c) Cs_2CO_3, DMF, KI, 80 °C, 3 d, 85%. d) $Pd(PPh_3)_4$, Na_2CO_3, THF/H_2O, 75 °C, O/N, 90%. e) H_2, $Pd(OH)_2$/C, K_2CO_3, THF, RT, 5 h, 95%. f) EDCI, HOBt, THF/$CHCl_3$, RT, 2 d, 69% g) CH_2Cl_2/MeOH, RT, 2 d, 94%. h) $Zn(OTf)_2$, Toluene/DMF, 105 ºC, O/N, 25%.

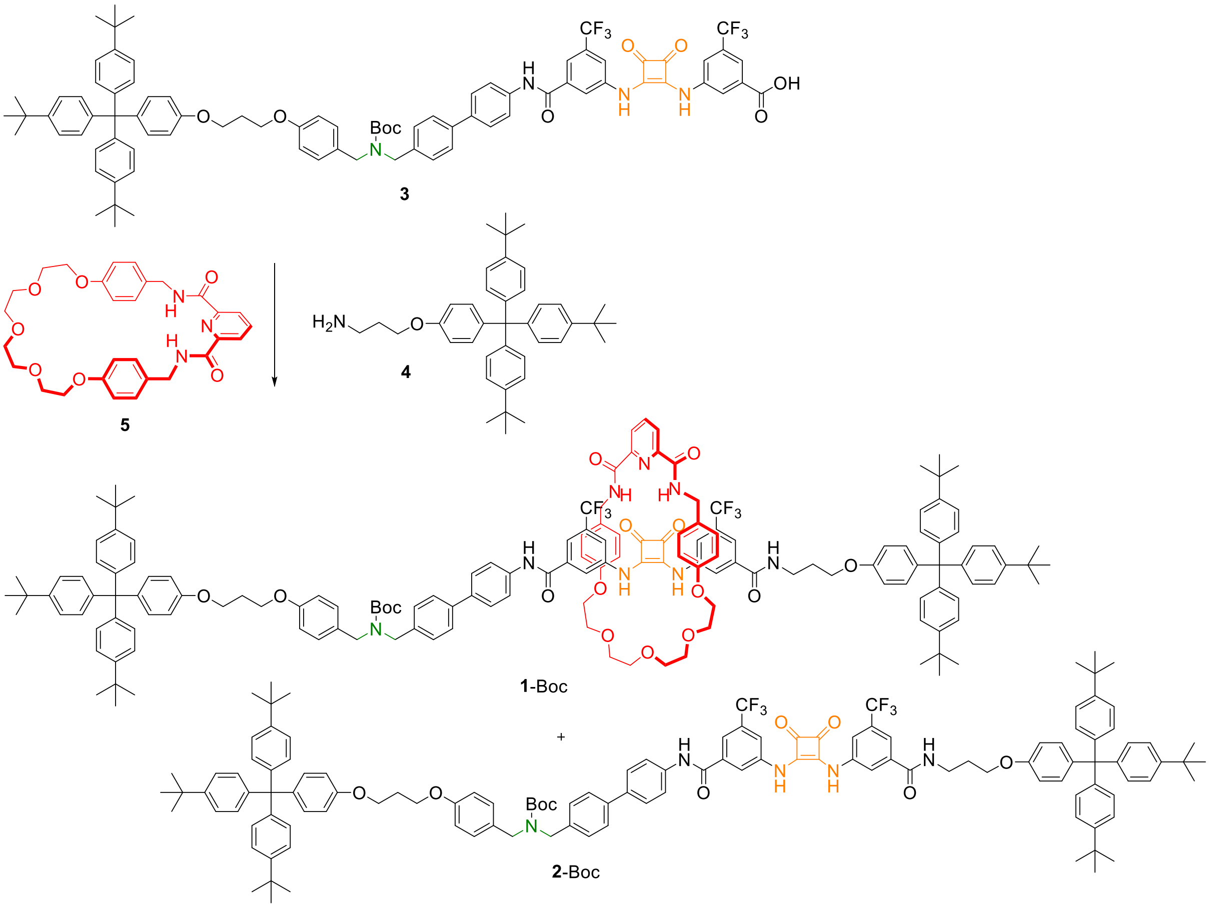

Scheme S2: Synthesis of rotaxane **1**-Boc. Reagents and conditions: PyBroP, DIPEA, CH_2Cl_2/THF/CH_3CN, RT, O/N, 47% (**1**-Boc), 46% (**2**-Boc).

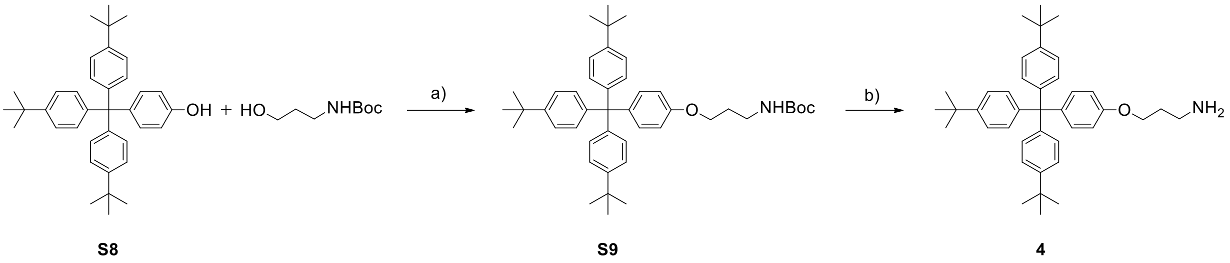

Scheme S3. Synthesis of amine functionalized stopper **4**. Reagents and conditions: a) PPh_3, DIAD, THF, RT, 12 h, 85%. b) CF_3CO_2H, CH_2Cl_2, RT, 4 h, 95%.

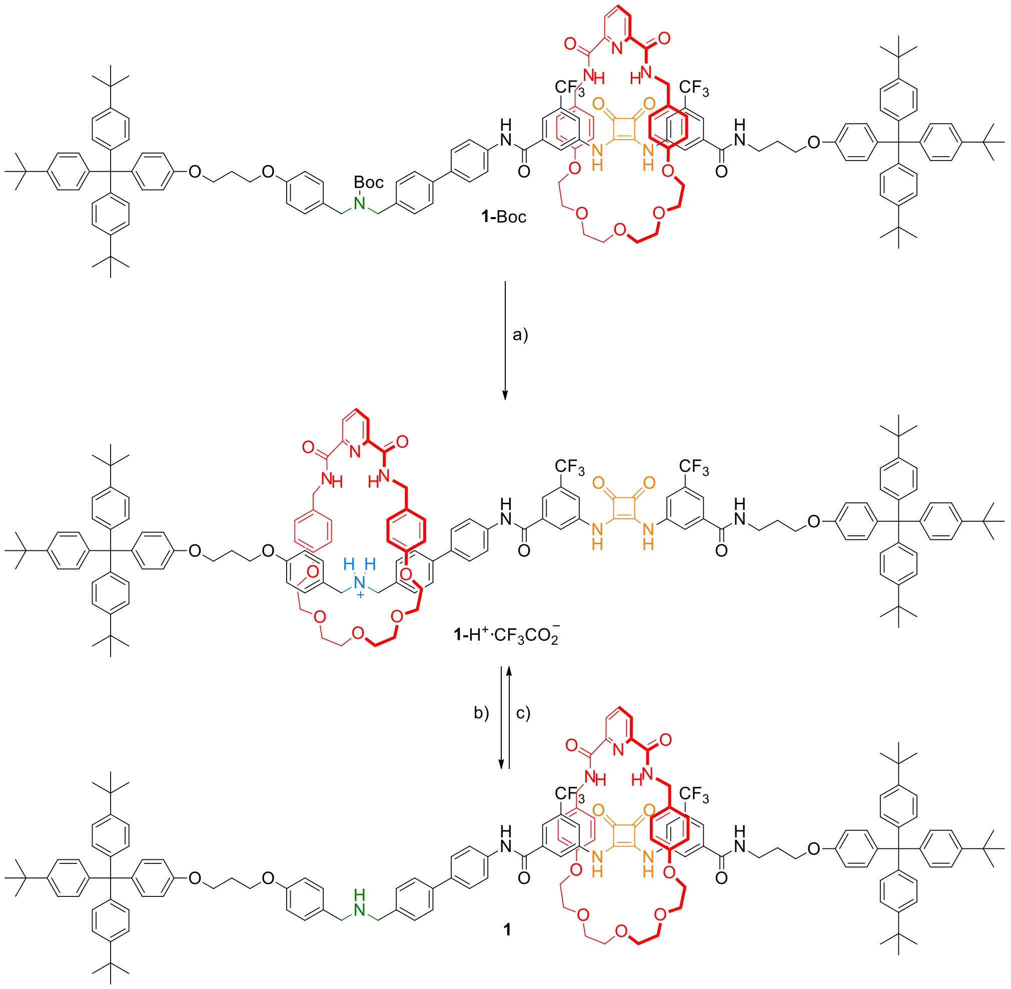

Scheme S4. Synthesis of rotaxane **1** and acid/base switching of the position of the macrocycle. Reagents and conditions: a) CF_3CO_2H, CH_2Cl_2, RT, 1.5 h, quant. b) $NaOH_{(aq)}$ 2M, CH_2Cl_2, RT, 1 h, quant. c) CF_3CO_2H, CH_2Cl_2, RT, 30 min, quant.

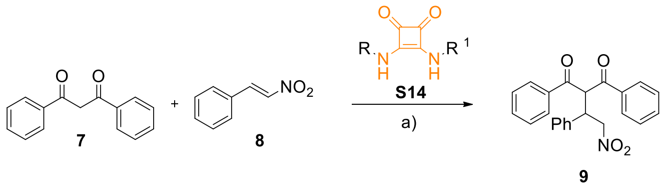

Scheme S5. Synthesis of compound **9**. Reagents and conditions: a) NaOAc, CH_2Cl_2, RT, 16 h, 70%.

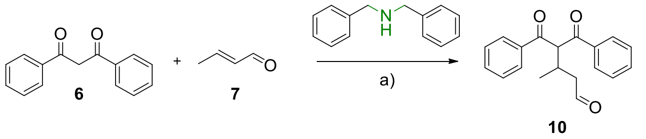

Scheme S6. Synthesis of compound **10**.[4] Reagents and conditions: a) CH_2Cl_2, RT, 48 h, 70%.

1.3. Synthetic Procedures and Characterization Details

Synthesis of S1

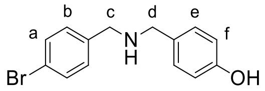

A solution of 4-hydroxybenzaldehyde (0.66 g, 5.37 mmol, 1.00 equiv.) and 4-bromobenzylamine (0.68 mL, 5.37 mmol, 1.00 equiv.) in dry MeOH (20 mL) was stirred overnight at room temperature under a N_2 atmosphere. The white precipitate formed was filtered and dissolved in THF/MeOH (1:3, 100 mL). To the resulting solution, $NaBH_4$ (0.609 g, 16.3 mmol, 3.00 equiv.) was added and the mixture was stirred overnight at room temperature under N_2. $NH_4Cl_{aq(sat)}$ (25 mL) was added and the organic solvents were removed under reduced pressure. The resulting mixture was extracted with EtOAc (3 × 150 mL). The combined organic extracts were washed with brine (200 mL), dried with $MgSO_4$ and concentrated under reduced pressure to afford **S1** (1.40 g, 89%) as a pale brown solid. M. p. 142−143 °C. ^{1}H NMR (400 MHz, CD_3OD) δ: 7.57 (d, J = 8.4 Hz, 2H, H_a), 7.38 (d, J = 8.5 Hz, 2H, H_b), 7.28 (d, J = 8.5 Hz, 2Hm H_e), 6.83 (d, J = 8.4 Hz, 2H, H_f), 4.05 (s, 2H, H_c), 4.01 (s, 2H, H_d). ^{13}C NMR (100 MHz, CD_3OD) δ: 159.43, 133.90, 133.15, 132.65, 132.24, 124.89, 123.98, 116.75, 52.20, 51.39. HRMS (ESI$^+$): m/z = 292.0336 [M+H]$^+$ (calcd. 292.0332 for $C_{14}H_{15}NOBr$).

Synthesis of S2

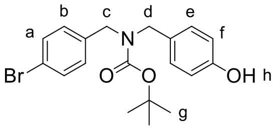

A solution of **S1** (1.38 g, 4.72 mmol, 1.00 equiv.), Boc_2O (1.29 g, 5.90 mmol, 1.25 equiv.) and Et_3N (10 mL) in MeOH (80 mL) was stirred overnight at room temperature. Water (30 mL) was added and the MeOH was removed under reduced pressure. The resulting mixture was extracted with CH_2Cl_2 (3 × 100 mL). The combined organic extracts were washed with water (150 mL), $NaHCO_{3(sat)}$ (150 mL), water (150 mL) and dried with $MgSO_4$. The solvent was removed under reduced pressure to yield **S2** (1.55 g, 84%) as a yellow oil which crystallized after several days. M. p. 122−123 °C. ^{1}H NMR (600 MHz, $CDCl_3$) δ: 7.44 (d, J = 7.9 Hz, 2H, H_a), 7.04 (br m, 4H, H_{b+e}), 6.78 (br, 2H, H_f), 5.79 (s, 1H, H_h), 4.33–4.24 (m, 4H, H_{c+d}), 1.50 (m, 9H, H_g). ^{13}C NMR (150 MHz, $CDCl_3$) δ: 156.24, 156.14, 155.39, 137.23, 136.99, 131.74, 129.79, 129.14, 121.59, 121.22, 121.11, 115.60, 80.69, 49.00, 48.83, 48.31, 28.60, 27.84.[5] HRMS (ESI$^+$): m/z = 414.0682 [M+Na]$^+$ (calcd. 414.0675 for $C_{19}H_{22}NO_3BrNa$).

Synthesis of S3

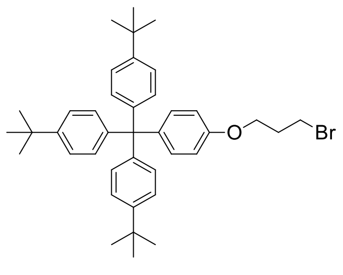

Synthesised according to a literature procedure.[2]

Synthesis of S4

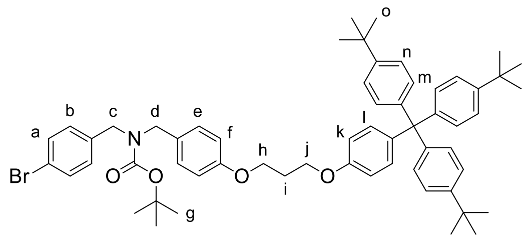

A mixture of **S2** (1.40 g, 3.57 mmol, 1.50 equiv.), **S3** (1.49 g, 2.38 mmol, 1.00 equiv.), Cs_2CO_3 (5.82 g, 17.85 mmol, 7.50 equiv.) and a catalytic amount of KI in dry DMF (50 mL) was stirred at 80 °C for 3 d under N_2. The solvent was removed under reduced pressure and the residue was partitioned between CH_2Cl_2 (750 mL) and water (100 mL). The layers were separated and the aqueous layer was extracted with CH_2Cl_2 (2 × 60 mL). The combined organic extracts were dried with $MgSO_4$ and the solvent removed under reduced pressure. Column chromatography (SiO_2, petrol ether/CH_2Cl_2 2:1) of the residue gave **S4** (1.90 g, 85%) as a colourless solid. M. p. 85−86 °C. ^{1}H NMR (600 MHz, $CDCl_3$) δ: 7.43 (d, J = 8.0 Hz, 2H, H_a), 7.23 (d, J = 8.2 Hz, 6H, H_n), 7.08 (m, 12H, $H_{b+e+m+l}$), 6.86 (d, J = 8.2 Hz, 2H, H_f), 6.78 (d, J = 8.5 Hz, 2H, H_k), 4.39–4.20 (br m, 4H, H_{c+d}), 4.14 (m, 4H, H_{h+j}), 2.25 (quin, J = 6.1 Hz, 2H, H_i), 1.49 (m, 9H, H_g), 1.30 (s, 27H, H_o). ^{13}C NMR (150 MHz, $CDCl_3$) δ: 158.37, 156.74, 156.01, 148.42, 144.25, 139.80, 137.22, 132.39, 131.71, 130.84, 129.87, 129.57, 129.17, 128.93, 124.17, 114.66, 113.06, 80.36, 64.63, 64.28, 63.17, 48.92, 48.67, 48.26, 34.43, 31.52, 29.47, 28.60. HRMS (ESI$^+$): m/z = 953.4831 $[M+NH_4]^+$ (calcd. 953.4826 for $C_{59}H_{74}N_2O_4Br$).

Synthesis of S5

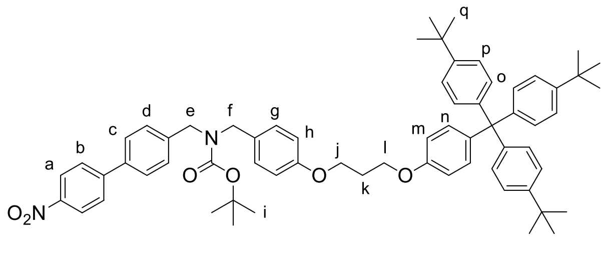

A solution of **S4** (1.89 g, 2.02 mmol, 1.00 equiv.) and 4-nitrophenylboronic acid (0.35 g, 2.12 mmol, 1.05 equiv) in THF/water (1:1, 300 mL) was purged with N_2 for 40 min. $Pd(PPh_3)_4$ (0.23 g, 0.20 mmol, 0.10 equiv) and Na_2CO_3 (1.28 g, 12.12 mmol, 6.00 equiv.) were added and the resulting mixture was stirred overnight at 75 °C under a N_2 atmosphere. CH_2Cl_2 (250 mL) was added and the layers separated. The aqueous layer was extracted with CH_2Cl_2 (2 × 100 mL) and the combined organic extracts were dried with $MgSO_4$ and concentrated under reduced pressure. The residue was purified by column chromatography (SiO_2, CH_2Cl_2/petrol ether 7:3 to CH_2Cl_2) to afford **S5** (1.78 g, 90%) as a yellow solid. M. p. 111−112 °C. ^{1}H NMR (600 MHz, $CDCl_3$) δ: 8.30 (d, J = 7.6 Hz, 2H, H_a), 7.73 (d, J = 8.2 Hz, 2H, H_b), 7.59 (d, J = 7.5 Hz, 2H, H_c), 7.40–7.27 (br, 2H, H_d), 7.23 (d, J = 6.8 Hz, 6H, H_p), 7.15 (br, 2H, H_g), 7.08 (m, 8H, H_{n+o}), 6.88 (d, J = 7.4 Hz, 2H, H_h), 6.78 (d, J = 7.8 Hz, 2H, H_m), 4.38 (br m, 4H, H_{e+f}), 4.15 (m, 4H, H_{j+l}), 2.25 (quin, J = 6.2 Hz, 2H, H_k), 1.52 (m, 9H, H_i), 1.30 (s, 27H, H_q). ^{13}C NMR (150 MHz, $CDCl_3$) δ: 158.39, 156.73, 156.08, 148.42, 147.39, 147.15, 144.25, 139.82, 139.32, 137.75, 132.40, 130.83, 129.94, 129.59, 128.95, 128.79, 128.26, 127.80, 127.67,

124.28, 124.18, 114.67, 113.06, 80.37, 64.64, 64.27, 63.17, 49.10, 48.67, 48.52, 34.43, 31.52, 29.48, 28.62. LRMS (ESI$^+$): *m/z* = 996.0 $[M+NH_4]^+$ (calcd. 996.6 for $C_{65}H_{78}N_3O_6$).

Synthesis of S6

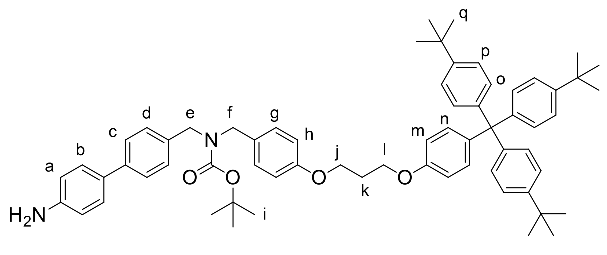

A solution of **S5** (1.70 g, 1.74 mmol, 1.00 equiv.) in THF (120 mL) was purged with N_2 for 20 min. $Pd(OH)_2/C$ (0.17 g, 10%w/w) and K_2CO_3 (1.20 g, 8.68 mmol, 5.00 equiv) were added and the resulting mixture was stirred under a H_2 atmosphere at room temperature for 5 h. The resulting mixture was filtered through a pad of Celite® and the filtrate was concentrated under reduced pressure. The residue was purified by column chromatography (SiO_2, CH_2Cl_2 to CH_2Cl_2/EtOAc 95:5) to yield **S6** (1.57 g, 95%) as a white solid. M. p. 105−106 °C. ^{1}H NMR (600 MHz, $CDCl_3$) δ: 7.49 (d, J = 8.1 Hz, 2H, H_c), 7.43 (d, J = 8.3 Hz, 2H, H_b), 7.23 (d, J = 8.5 Hz, 6H, H_p), 7.21–7.10 (br, 4H, H_{d+g}), 7.09 (m, 8H, H_{n+o}), 6.87 (d, J = 8.5 Hz, 2H, H_h), 6.83 (d, J = 8.3 Hz, 2H, H_a), 6.78 (d, J = 8.9 Hz, 2H, H_m), 4.35 (br, 4H, H_{e+f}), 4.14 (m, 4H, H_{j+l}), 2.26 (quin, J = 6.1 Hz, 2H, H_k), 1.51 (s, 9H, H_i), 1.30 (s, 27H, H_q). ^{13}C NMR (150 MHz, $CDCl_3$) δ: 158.28, 156.76, 156.13, 148.42, 144.63, 144.51, 144.26, 140.07, 139.78, 136.25, 132.39, 130.85, 130.22, 129.58, 128.96, 128.57, 128.10, 127.98, 126.64, 124.17, 116.18, 114.61, 113.07, 80.13, 64.62, 64.31, 63.17, 48.77, 48.37, 34.43, 31.53, 29.49, 28.65. LRMS (ESI$^+$): *m/z* = 949.3 $[M+H]^+$ (calcd. 949.6 for $C_{65}H_{77}N_2O_4$).

Synthesis of S7

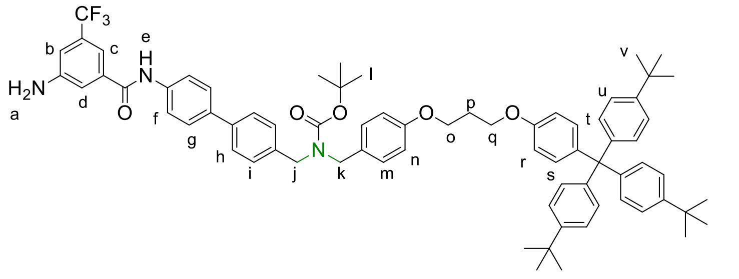

To a solution of **S6** (0.40 g, 0.42 mmol, 1.00 equiv.) and 3-amino-5-trifluoromethyl-benzenecarboxylic acid (87 mg, 0.42 mmol, 1.00 equiv.) in THF/$CHCl_3$ (3:1, 8 mL), HOBt (71 mg, 0.46 mmol, 1.10 equiv.) and EDCI (89 mg, 0.46 mmol, 1.10 equiv.) were added and the resulting mixture was stirred at room temperature for 2 d, under a N_2 atmosphere. The solvent was removed under reduced pressure and the residue was purified by column chromatography (SiO_2, CH_2Cl_2/EtOAc 95:5) to yield **S7** (0.33 g, 69%) as a pale brown solid. ^{1}H NMR (600 MHz, $CDCl_3$) δ: 7.79 (s, 1H, H_e), 7.71 (d, J = 8.7 Hz, 2H, H_f), 7.62 (d, J = 8.3 Hz, 2H, H_g), 7.55 (d, J = 8.0 Hz, 2H, H_o), 7.38 (s, 1H, $H_{c/d}$), 7.37 (s, 1H, $H_{c/d}$), 7.29 (m, 2H, H_i), 7.22 (d, J = 8.5 Hz, 6H, H_u), 7.20–7.10 (m, 2H, H_m), 7.10–7.06 (m, 8H, H_{s+t}), 7.06 (s, 1H, H_b), 6.87 (d, J = 8.5 Hz, 2H, H_n), 6.78 (d, J = 8.8 Hz, 2H, H_r), 4.36 (m, 4H, H_{j+k}), 4.14 (m, 4H, H_{o+q}), 2.25 (quin, J = 5.9 Hz, 2H, H_p), 1.51 (s, 9H, H_l), 1.29 (s, 27H, H_v). ^{13}C NMR (150 MHz,

$CDCl_3$) δ: 164.75, 158.33, 156.76, 156.15, 148.43, 147.64, 144.27, 139.80, 139.41, 137.11, 132.40 (q, J = 32.5 Hz), 132.39, 130.84, 130.14, 129.59, 128.97, 128.62, 128.06, 127.78, 127.11, 124.60 (q, J = 272.6 Hz), 124.18, 120.74, 120.64, 116.86, 114.64, 114.40, 113.08, 112.71, 80.24, 64.64, 64.32, 63.18, 48.85, 48.49, 34.44, 31.52, 29.49, 28.65. LRMS (ESI^-): m/z = 1170.7 $[M+Cl]^-$ (calcd. 1170.6 for $C_{73}H_{80}F_3N_3O_5Cl$).

Synthesis of S8

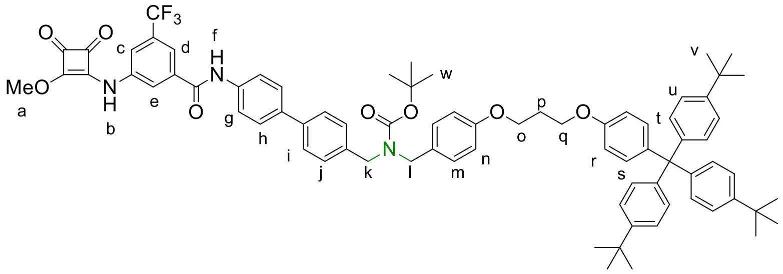

A mixture of **S7** (0.13 g, 0.12 mmol, 1.00 equiv.), 3,4-dimethoxycyclobut-3-ene-1,2-dione (33 mg, 0.23 mmol, 2.00 equiv.) and zinc triflate (17 mg, 0.048 mmol, 0.40 equiv.) in CH_2Cl_2/MeOH (1:1, 3 mL) was stirred at room temperature for 2 d, under a N_2 atmosphere. The resulting mixture was concentrated under reduced pressure and the residue purified by column chromatography (SiO_2, CH_2Cl_2/EtOAc 95:5 to 85:15) to afford **S8** (0.14 g, 94%) as a pale yellow solid. ^{1}H NMR (600 MHz, CD_3COCD_3) δ: 9.87 (s, 1H, H_f), 8.33 (s, 1H, H_e), 8.10 (s, 1H, H_c), 8.04 (s, 1H, H_d), 7.93 (d, J = 8.7 Hz, 2H¸ H_g), 7.69 (d, J = 8.6 Hz, 2H, H_n), 7.64 (d, J = 7.8 Hz, 2H, H_i), 7.34 (m, 2H, H_j), 7.29 (d, J = 8.6 Hz, 6H, H_u), 7.21 (m, 2H, H_m), 7.12 (d, J = 8.5 Hz, 6H, H_t), 7.08 (d, J = 8.8 Hz, 2H, H_s), 6.93 (d, J = 8.1 Hz, 2H, H_n), 6.84 (d, J = 8.9 Hz, 2H, H_r), 4.50 (s, 3H, H_a), 4.46–4.30 (m, 4H, H_{l+k}), 4.17 (m, 4H, H_{o+q}), 2.23 (quin, J = 6.2 Hz, 2H, H_p), 1.49 (s, 9H, H_w), 1.29 (s, 27H, H_v). 13C NMR (150 MHz, CD_3COCD_3) δ: 188.20, 185.55, 180.65, 170.21, 164.40, 159.21, 157.81, 156.32, 149.10, 145.23, 140.56, 140.23, 140.04, 139.22, 139.13, 138.58, 138.55, 137.24, 132.79, 132.12 (q, J = 32.6 Hz), 131.40, 131.20, 130.12, 129.76, 129.23, 128.89, 127.91, 127.47, 125.03, 124.70 (q, J = 272.6 Hz), 122.93, 121.46, 121.37, 119.57, 119.31, 115.31, 114.02, 80.10, 65.17, 65.01, 63.86, 61.50, 49.59, 49.18, 34.83, 31.64, 30.05, 28.60. LRMS (ESI^-): m/z = 1244.7 $[M-H]^-$ (calcd. 1244.6 for $C_{78}H_{81}F_3N_3O_8$).

Synthesis of 3

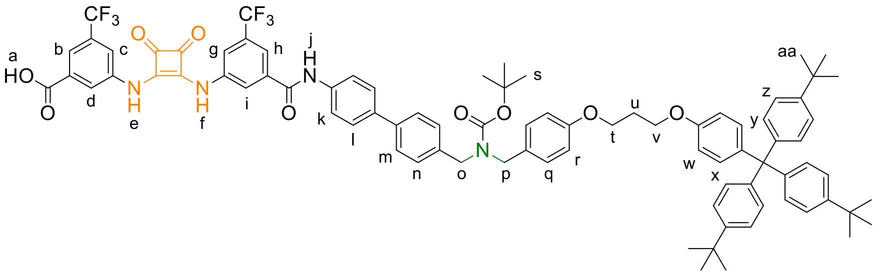

A solution of **S8** (50 mg, 0.040 mmol), 3-amino-5-trifluoromethyl-benzenecarboxylic acid (12 mg, 0.06 mmol) and $Zn(OTf)_2$ (4.4 mg, 0.012 mmol) in a dry Toluene/DMF mixture (20:1, 3.65 mL) was stirred at 105 ºC overnight. The solvent was removed under reduced

pressure and the residue was purified by preparative thin layer chromatography (SiO_2, CH_2Cl_2:MeOH 92:8) to yield **3** (14 mg, 25%) as a pale yellow solid. ^{1}H NMR (600 MHz, CD_3COCD_3) δ: 13.38 (s, 1H, H_a), 10.83 (s, 1H, $H_{e/f}$), 10.21 (m, 2H, $H_{e/f+Ar}$), 8.96 (s, 1H, H_{Ar}), 8.78 (s, 1H, H_{Ar}), 8.47 (s, 1H, H_{Ar}), 8.38 (s, 1H, H_{Ar}), 8.30 (d, J = 8.2 Hz, 2H, H_k), 7.99 (s, 1H, H_{Ar}), 7.72 (d, J = 8.1 Hz, 2H, H_l), 7.68 (d, J = 7.8 Hz, 2H, H_m), 7.37 (br, 2H, H_n), 7.31 (d, J = 8.2 Hz, 6H, H_z), 7.25 (br, 2H, H_q), 7.12 (d, J = 8.2 Hz, 6H, H_y), 7.09 (d, J = 8.6 Hz, 2H, H_x), 6.96 (d, J = 8.1 Hz, 2H, H_r), 6.87 (d, J = 8.5 Hz, 2H, H_w), 4.53–4.31 (m, 4H, H_{o+p}), 4.20 (m, 4H, H_{t+v}), 2.25 (quin, J = 5.9 Hz, 2H, H_u), 1.51 (s, 9H, H_s), 1.30 (s, 27H, H_{aa}). ^{13}C NMR (150 MHz, CD_3COCD_3) δ: 183.49, 182.63, 170.26, 167.63, 167.39, 164.06, 159.22, 157.85, 156.35, 149.11, 145.26, 142.67, 142.06, 140.87, 140.37, 140.23, 139.92, 138.22, 136.94, 136.75, 132.79, 132.54, 131.40, 131.32, 130.21, 129.82, 129.29, 128.95, 127.85, 127.48, 126.39, 126.08, 125.05, 124.59, 124.28, 123.76, 122.59, 122.01, 121.23, 121.15, 118.78, 118.35, 117.92, 115.32, 114.05, 80.07, 65.18, 65.05, 63.87, 49.15, 34.85, 31.64, 29.65,[6] 28.62. LRMS (ESI^-): m/z = 1417.8 $[M-H]^-$ (calcd. 1417.6 for $C_{85}H_{83}F_6N_4O_9$).

Synthesis of 4

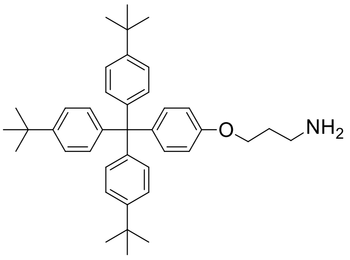

To a solution of **S9** (1.11 g, 1.68 mmol, 1.00 equiv.) in CH_2Cl_2 (9.5 mL), CF_3CO_2H (2.5 mL) was added dropwise. The resulting mixture was stirred in an open flask for 4 h. $NaHCO_{3aq(sat)}$ was added in portions until pH>9. The mixture was extracted with CH_2Cl_2 (3 × 15 mL). The combined organic extracts were washed with H_2O (20 mL) and brine (20 mL) and dried with $MgSO_4$. The solvent was removed under reduced pressure to afford **4** (0.90 g, 95%) as a white solid. The characterization data are in agreement with those previously reported.[7]

Synthesis of 5

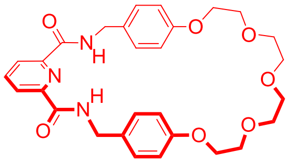

Synthesized according to a literature procedure.[3]

Synthesis of 1-Boc and 2-Boc

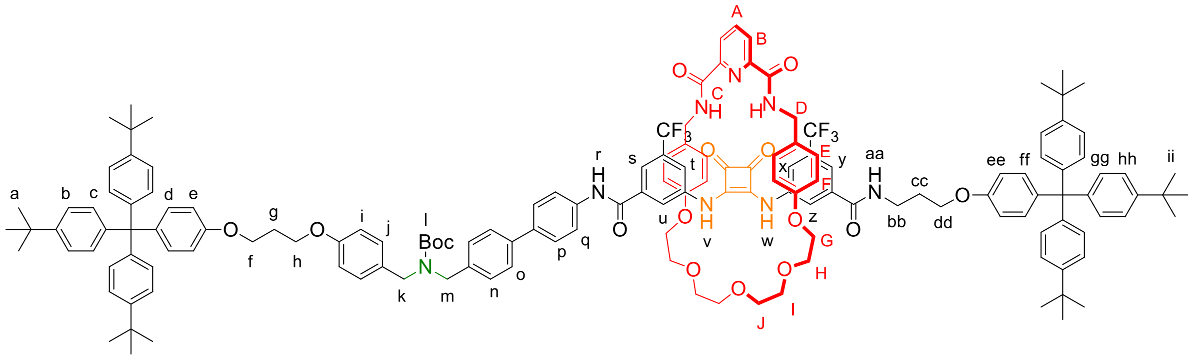

3 (30.0 mg, 0.021 mmol, 1.00 equiv), **5** (26.0 mg, 0.050 mmol, 2.35 equiv.) and **4** (30 mg, 0.055 mmol, 2.60 equiv) were dissolved in a mixture of CH_2Cl_2/THF/CH_3CN (60:35:5, 3.6 mL). The solution was stirred under N_2 atmosphere, at room temperature for 1 h after which PyBroP (20.0 mg, 0.043 mmol, 2.00 equiv.) and *N*,*N*-diisopropylethylamine (10 µL, 0.050 mmol, 2.35 equiv.) were added and the mixture stirred at room temperature for additional 20 h. The solvents were removed under reduced pressure and the crude was purified by column chromatography (SiO_2, CH_2Cl_2:MeOH 98:2) to afford **1**-Boc (25 mg, 47%) as a colourless solid. ^{1}H NMR (600 MHz, CD_2Cl_2) δ: 10.23 (s, 1H, $H_{v/w}$), 10.13 (s, 1H, $H_{v/w}$), 9.15 (t, J = 5.7 Hz, 2H, H_C), 8.55 (s, 1H, H_r), 8.44 (d, J = 7.8 Hz, 2H, H_B), 8.40 (s, 1H, $H_{s/u}$), 8.13 (s, 1H, $H_{y/z}$), 8.08 (t, J = 7.9 Hz, 1H, H_A), 8.04 (s, 1H, $H_{s/u}$), 7.95 (s, 1H, H_x), 7.80 (s, 2H, $H_{t+y/z}$), 7.78 (d, J = 8.5 Hz, 2H, H_q), 7.62 (m, 4H, H_{o+p}), 7.38–7.33 (m, 2H, H_n), 7.32–7.26 (m, 12H, H_{b+hh}), 7.24–7.17 (m, 16H, $H_{c+gg+d+ff+j}$), 6.94 (t, J = 7.9 Hz, 6H, H_{i+E}), 6.85 (d, J = 3.3 Hz, 2H, $H_{e/ee}$), 6.84 (d, J = 3.3 Hz, 2H, $H_{e/ee}$), 6.30 (d, J = 8.4 Hz, 4H, H_F), 4.56–4.45 (m, 4H, H_D), 4.41 (m, 4H, H_{k+m}), 4.19 (m, 4H, H_{f+h}), 4.13 (t, J = 5.7 Hz, 2H, H_{dd}), 3.90 – 3.80 (m, 16H, $H_{G+H+I+J}$), 3.68 (q, J = 6.3 Hz, 2H, H_{bb}), 2.28 (quin, J = 6.1 Hz, 2H, H_g), 2.13 (quin, J = 6.3 Hz, 2H, H_{cc}), 1.55 (s, 9H, H_l), 1.34 (s, 27H, $H_{a/ii}$), 1.33 (s, 27H, $H_{a/ii}$). ^{13}C NMR (150 MHz, CD_2Cl_2) δ: 181.81, 181.45, 165.14, 163.80, 163.71, 163.31, 158.24, 157.27, 156.73, 156.44, 155.79, 149.43, 148.44, 148.38, 144.50, 144.46, 140.13, 139.73, 139.58, 139.36, 139.11, 138.34, 137.35, 137.04, 136.98, 131.98, 131.88, 130.99, 130.38, 130.34, 129.94, 127.53, 126.82, 124.66, 124.31, 124.28, 122.68, 120.43, 120.13, 120.02, 119.27, 118.19, 117.73, 114.41, 113.71, 113.15, 113.12, 79.80, 70.74, 70.51, 70.20, 66.91, 66.22, 64.51, 64.34, 63.09, 63.08, 48.84, 48.35, 42.88, 42.75, 38.29, 34.18, 31.59, 31.07, 30.94, 29.32, 28.95, 28.19.

2-Boc (19 mg, 46%) was also isolated as a colourless solid.

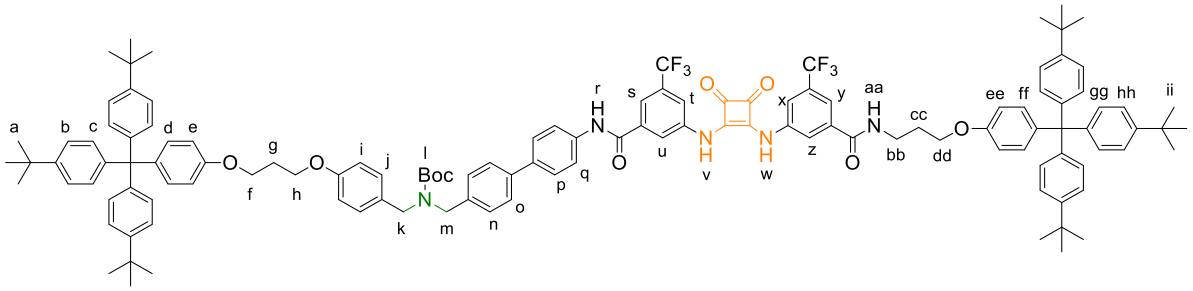

^{1}H NMR (600 MHz, CD_3COCD_3) δ: 10.11 (s, 1H, $H_{v/w}$), 9.87 (s, 1H, $H_{v/w}$), 8.30 (s, 1H, H_{Ar}), 8.23 (s, 1H, H_{Ar}), 8.20 (s, 3H, H_{Ar}), 7.98–7.87 (m, 2H, H_q), 7.82 (s, 1H, H_{Ar}), 7.64 (dd, J = 15.9,

7.9 Hz, 4H, H_{o+p}), 7.36–7.27 (m, 14H, H_{b+n+hh}), 7.26–7.19 (m, 2H, H_j), 7.14 (m, 12H, H_{c+gg}), 7.11–7.07 (m, 4H, H_{d+ff}), 6.95 (d, J = 8.2 Hz, 2H, H_i), 6.86 (d, J = 8.8 Hz, 2H, $H_{e/ee}$), 6.80 (d, J = 8.4 Hz, 2H, $H_{e/ee}$), 4.39 (m, 4H, H_{k+m}), 4.25–4.15 (m, 4H, H_{f+h}), 4.06 (t, J = 6.0 Hz, 2H, H_{dd}), 3.69 – 3.56 (m, 2H, H_{bb}), 2.25 (quin, J = 6.2 Hz, 2H, H_g), 1.51 (s, 9H, H_l), 1.31 (s, 27H, $H_{a/ii}$), 1.30 (s, 27H, $H_{a/ii}$). ^{13}C NMR (150 MHz, CD_3COCD_3) δ: 182.76, 182.71, 166.08, 165.91, 164.83, 158.36, 156.97, 156.95, 155.46, 148.25, 148.20, 144.41, 144.39, 140.23, 140.03, 139.37, 139.31, 139.21, 138.31, 137.43, 137.13, 136.34, 131.94, 131.88, 130.55, 130.52, 130.35, 129.30, 128.91, 128.34, 128.01, 127.01, 126.63, 124.76, 124.61, 124.19, 122.95, 122.91, 121.63, 121.36, 120.59, 118.26, 117.95, 117.80, 114.46, 113.17, 113.12, 79.22, 65.13, 64.31, 64.16, 63.00, 48.69, 48.28, 36.97, 33.97, 31.42, 27.75. LRMS (ESI$^+$): m/z = 1980.8 $[M+NH_4]^+$ (calcd. 1980.0 for $C_{125}H_{137}F_6N_6O_9$).

Synthesis of S8

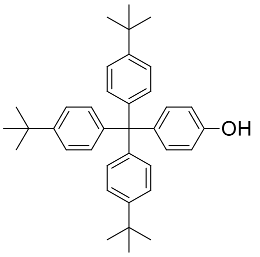

Synthesised according to a literature procedure.[1]

Synthesis of S9

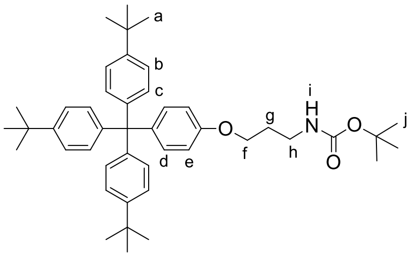

To a solution of **S8** (1.00 g, 1.98 mmol, 1.00 equiv.) in dry THF (20 mL), *tert*-butyl-3-hydroxypropylcarbamate (0.69 g, 3.96 mmol, 2.00 equiv.) and PPh_3 (1.04 g, 3.96 mmol, 2.00 equiv.) were added and the resulting mixture was cooled in a water-ice bath. DIAD (0.80 g, 3.96 mmol, 2.00 equiv.) was added and the resulting mixture was stirred overnight at room temperature under a N_2 atmosphere. The solvent was removed under reduced pressure and the crude mixture was dissolved in CH_2Cl_2 (3 mL). MeOH (100 mL) was added and the colourless precipitate formed was filtered off and dried under vacuum to give **S9** (1.11 g, 1.68 mmol, 85%) as a colourless solid. M. p. 126–127 °C. ^{1}H NMR (600 MHz, $CDCl_3$) δ: 7.23 (d, J = 8.2 Hz, 6H, H_b), 7.08 (d, J = 8.3 Hz, 8H, H_{c+d}), 6.76 (d, J = 8.5 Hz, 2H, H_e), 4.79, (s, 1H, H_i) 4.00 (t, J = 5.9 Hz, 2H, H_f), 3.35–3.28 (m, 2H, H_h), 2.00–1.92 (m, 2H, H_g), 1.44 (s, 9H, H_j), 1.30 (s, 27H, H_a). ^{13}C NMR (150 MHz, $CDCl_3$) δ: 156.66, 156.14, 148.43, 144.24, 139.89, 132.39, 130.84, 124.18, 113.04, 79.32, 65.87, 63.17, 38.34, 34.44, 31.53, 29.66, 28.56. HRMS (ESI$^+$): m/z = 684.4391 $[M+Na]^+$ (calcd. 684.4387 for $C_{45}H_{59}NO_3Na$).

Synthesis of 1-H$^+$·CF$_3$CO$_2^-$

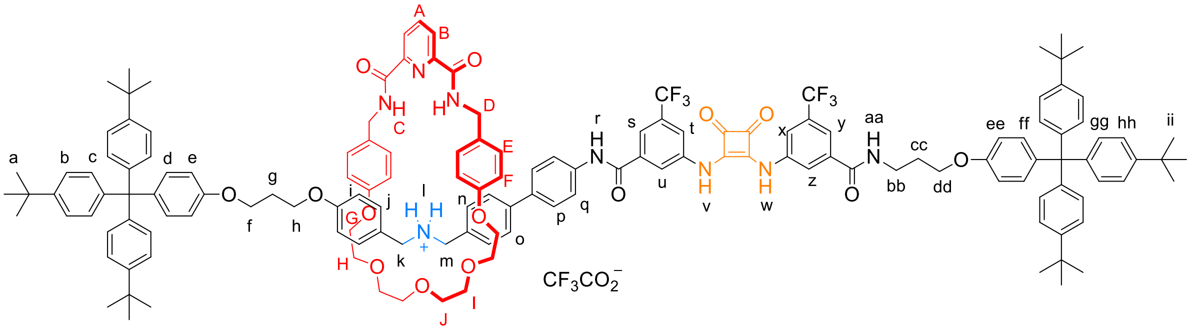

Procedure A: From **1**-Boc

Rotaxane **1**-Boc (25.0 mg, 0.010 mmol, 1.00 equiv.) was dissolved in CH_2Cl_2 (1.0 mL). CF_3CO_2H (0.25 mL) was added and the mixture was stirred at room temperature for 90 min. The solvents were removed under reduced pressure to yield **1**-H$^+$·CF$_3$CO$_2^-$ (25 mg, quant.) as a solid which was used in the next step without further purification.

Procedure B: From **1**

1 (22 mg, 0.092 mmol, 1.00 equiv.) was dissolved in CH_2Cl_2 (1.0 mL). CF_3CO_2H (10 µL, 0.13 mmol, 1.4 equiv.) was added and the mixture was stirred at room temperature for 30 min. The solvents were removed under reduced pressure to yield **1**-H$^+$·CF$_3$CO$_2^-$ (23 mg, 0.092 mmol, quant.) as a solid.

^{1}H NMR (600 MHz, CD_2Cl_2) δ: 10.22 (s, 1H, $H_{v/w}$), 10.11 (s, 1H, $H_{v/w}$), 9.14 (t, J = 5.8 Hz, 2H, H_C), 8.53 (s, 1H, H_r), 8.43 (d, J = 7.8 Hz, 2H, H_B), 8.40 (s, 1H, $H_{s/u}$), 8.13 (s, 1H, $H_{y/z}$), 8.08 (t, J = 7.9 Hz, 1H, H_A), 8.03 (s, 1H, $H_{s/u}$), 7.94 (s, 1H, H_x), 7.80 (s, 2H, $H_{t+y/z}$), 7.79–7.75 (m, 2H, H_q), 7.62 (dd, J = 8.1, 4.8 Hz, 4H, H_{o+p}), 7.48 (d, J = 7.8 Hz, 2H, H_n), 7.33–7.26 (m, 14H, H_{b+hh+j}), 7.24–7.17 (m, 16H, $H_{c+gg+d+ff}$), 6.93 (dd, J = 11.3, 8.1 Hz, 6H, H_{i+E}), 6.88–6.81 (m, 4H, H_{e+ee}), 6.30 (d, J = 8.1 Hz, 4H, H_F), 4.51 (t, J = 5.9 Hz, 4H, H_D), 4.18 (m, 4H, H_{f+h}), 4.13 (t, J = 5.7 Hz, 2H, H_{dd}), 3.90 – 3.81 (m, 18H, $H_{k/m+G+H+I+J}$), 3.80 (s, 2H, $H_{k/m}$), 3.67 (m, 2H, H_{bb}), 2.28 (quin, J = 6.1 Hz, 2H, H_g), 2.13 (quin, J = 6.4 Hz, 2H, H_{cc}), 1.34 (s, 27H, $H_{a/ii}$), 1.33 (s, 27H, $H_{a/ii}$). ^{13}C NMR (150 MHz, CD_2Cl_2) δ: 181.82, 181.44, 165.28, 164.55, 163.87, 163.55, 159.92, 157.33, 156.64, 156.43, 149.26, 148.38, 148.36, 144.47, 144.46, 141.36, 140.13, 139.77, 139.58, 139. 36, 138.43, 136.97, 136.93, 136.01, 131.97, 131.89, 131.52, 130.37, 130.34, 129.92, 128.96, 128.86, 128.83, 128.68, 128.54, 128.34, 127.49, 127.27, 124.78, 124.31, 124.28, 122.67, 120.39, 120.32, 119.15, 118.13, 117.90, 117.84, 114.96, 113.74, 113.11, 70.73, 70.47, 70.17, 66.91, 66.24, 64.59, 64.16, 63.09, 63.07, 46.08, 42.98, 42.36, 38.35, 34.18, 31.94, 31.92, 31.07, 30.63, 29.70, 29.16.

Synthesis of 1

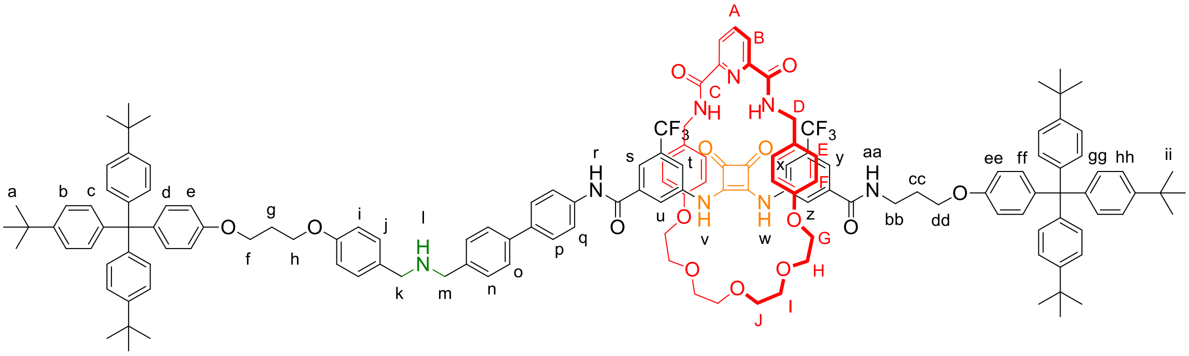

1-H$^+$·$CF_3CO_2^-$ (25 mg, 0.010 mmol, 1.00 equiv) was dissolved in CH_2Cl_2 (2.0 mL). $NaOH_{(aq)}$ 2M (2.0 mL) was added and the mixture was stirred at room temperature for 1 h. The layers were separated and the organic phase was dried with $MgSO_4$, filtered and the solvent removed under reduced pressure to yield **1** (22 mg, 92%) as a colourless oil which was used without further purification.

Synthesis of 2-H$^+$·$CF_3CO_2^-$

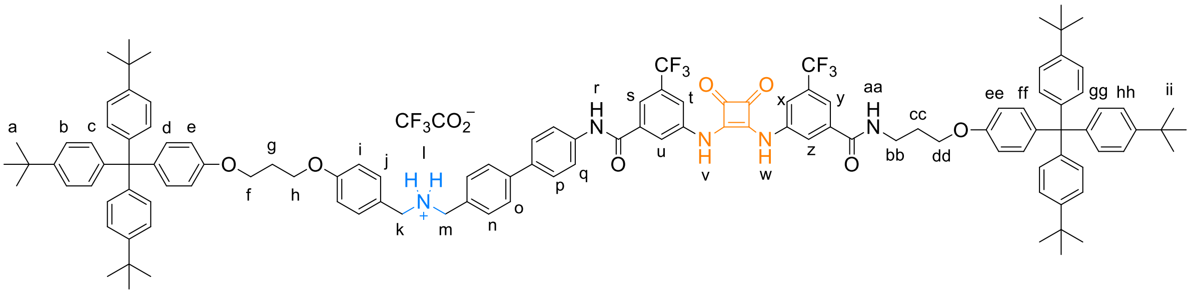

The free thread **2**-Boc (19 mg, 0.010 mmol, 1.00 equiv.) was dissolved in CH_2Cl_2 (1 mL). CF_3CO_2H (0.25 mL) was then added and the mixture was stirred at room temperature for 90 min. The mixture was concentrated to afford **2**-H$^+$·$CF_3CO_2^-$ (19 mg, quant.) as a solid which was used in the next step without further purification. ^{1}H NMR (600 MHz, CD_3COCD_3) δ: 9.74 (br s, 2H, $H_{v/w}$), 8.87 (s, 1H, H_s), 8.57 (s, 1H, H_y), 8.44 (s, 1H, $H_{t/x}$), 8.22 (s, 1H, $H_{t/x}$), 7.99 (d, J = 8.5 Hz, 2H, H_q), 7.94 (s, 1H, H_u), 7.86 (s, 1H, H_z), 7.68 (d, J = 8.0 Hz, 2H, H_n), 7.62 (d, J = 8.7 Hz, 2H, H_p), 7.59 (dd, J = 8.5, 6.7 Hz, 4H, H_{o+j}), 7.34–7.28 (m, 12H, H_{b+hh}), 7.15–7.10 (m, 12H, H_{c+gg}), 7.09 (d, J = 8.8 Hz, 4H, H_{d+ff}), 7.02 (d, J = 8.6 Hz, 2H, H_i), 6.86 (d, J = 8.9 Hz, 2H, $H_{e/ee}$), 6.83 (d, J = 8.9 Hz, 2H, $H_{e/ee}$), 4.59 (s, 2H, $H_{k/m}$), 4.52 (s, 2H, $H_{k/m}$), 4.22 (t, J = 6.2 Hz, 2H, $H_{f/h}$), 4.15 (m, 4H, $H_{f/h+dd}$), 3.70 (t, J = 6.8 Hz, 2H, H_{bb}), 2.24 (quin, J = 6.1 Hz, 3H, H_g), 2.19–2.13 (m, 2H, H_{cc}), 1.31 (s, 27H, $H_{a/ii}$), 1.29 (s, 29H, $H_{a/ii}$). ^{13}C NMR (150 MHz, CD_3COCD_3) δ: 181.88, 181.79, 165.81, 165.50, 163.03, 159.89, 157.02, 156.92, 148.28, 148.21, 144.40, 144.36, 141.09, 140.72, 140.61, 139.42, 139.36, 139.06, 136.94, 136.47, 134.65, 131.95, 131.92, 131.72, 130.78, 130.55, 130.52, 130.08, 128.87, 128.15, 126.78, 126.65, 125.23, 124.97, 124.87, 124.17, 123.46, 121.33, 121.01, 120.16, 118.31, 117.55, 117.38, 114.81, 113.18, 113.14, 65.16, 64.51, 64.07, 63.01, 50.89, 50.77, 36.97, 33.96, 30.80, 30.76.

Synthesis of 9

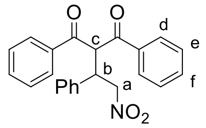

To a solution of *trans*-β-nitrostyrene (**8**) (74.6 mg, 0.50 mmol, 2.00 equiv.), 1,3-diphenylpropane-1,3-dione (**6**) (56.0 mg, 0.25 mmol, 1.00 equiv.) and **S14** (25.7 mg, 0.025 mmol, 0.10 equiv) in CH_2Cl_2/isopropanol (5:1, 0.6 mL), sodium acetate (4.0 mg, 0.05 mmol, 0.20 equiv.) was added and the mixture was stirred O/N at room temperature. The solvents were removed under reduced pressure and the crude residue was purified by column chromatography (SiO_2, petrol ether/EtOAc, 8:2) to yield **9** (65 mg, 70%) as a colourless solid. Characterisation data in agreement with previously reported compound.[8] ^{1}H NMR (600 MHz, $CDCl_3$) δ: 7.86 (dd, J = 8.3, 1.3 Hz, 2H, $H_{d/d'}$), 7.78 (dd, J = 8.3, 1.3 Hz, 2H, $H_{d/d'}$), 7.55 (t, J = 7.4 Hz, 1H, $H_{f/f'}$), 7.51 (t, J = 7.4 Hz, 1H, $H_{f/f'}$), 7.41–7.35 (m, 4H, $H_{e+e'}$), 7.25–7.15 (m, 5H, H_{Ar}), 5.84 (d, J = 8.0 Hz, 1H, H_c), 5.00 (d, J = 6.8 Hz, 2H, H_a), 4.62 (q, J = 7.1 Hz, 1H, H_b).

Synthesis of 10

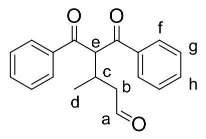

Synthesised according to a literature procedure.[4]

General procedure for catalytic tests

The catalyst (2.8 μmol, 0.05 equiv., 7 mg in case of **1**-$H^+ \cdot CF_3CO_2^-$, 6.7 mg in case of **1**, 3 mg in case of **S14**), *trans*-β-nitrostyrene (8.9 mg, 60 μmol, 1.00 equiv.), crotonaldehyde (10 μL, 120 μmol, 2.00 equiv.) and 1,3-diphenylpropane-1,3-dione (13.3 mg, 60 μmol, 1.00 equiv.) were dissolved in CH_2Cl_2 (0.12 mL) (with **1**, **1**-$H^+ \cdot CF_3CO_2^-$ or **2**-$H^+ \cdot CF_3CO_2^-$). Sodium acetate (0.5 mg, 6.0 μmol, 0.1 equiv.) was added and the mixture was stirred at room temperature. The progress of the reaction was monitored by taking 5 μL aliquots of the mixture, diluting by 0.5 mL of $CDCl_3$ and analysing by ^{1}H NMR spectroscopy.

1.4. ^{1}H NMR spectra for selected compounds

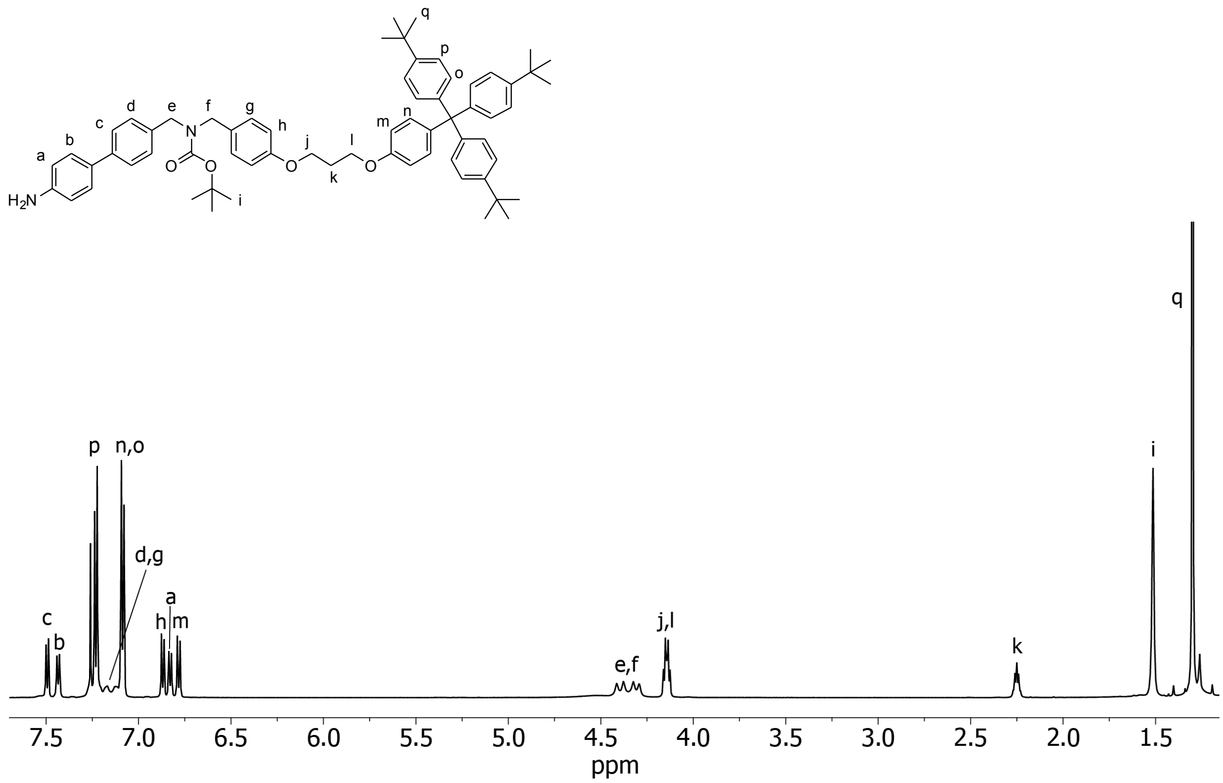

Figure S1. ^{1}H NMR spectrum (600 MHz, $CDCl_3$) of compound **S6**.

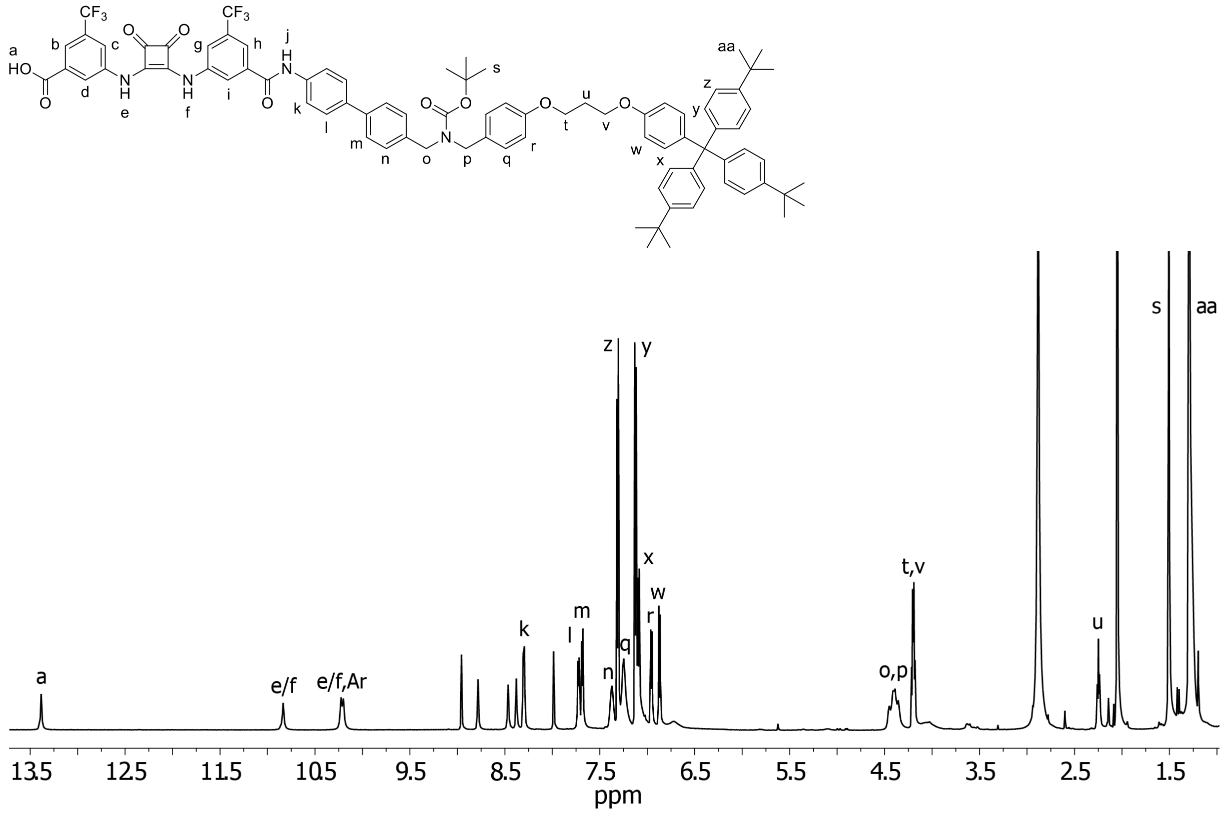

Figure S2. ^{1}H NMR spectrum (600 MHz, CD_3COCD_3) of compound **3**.

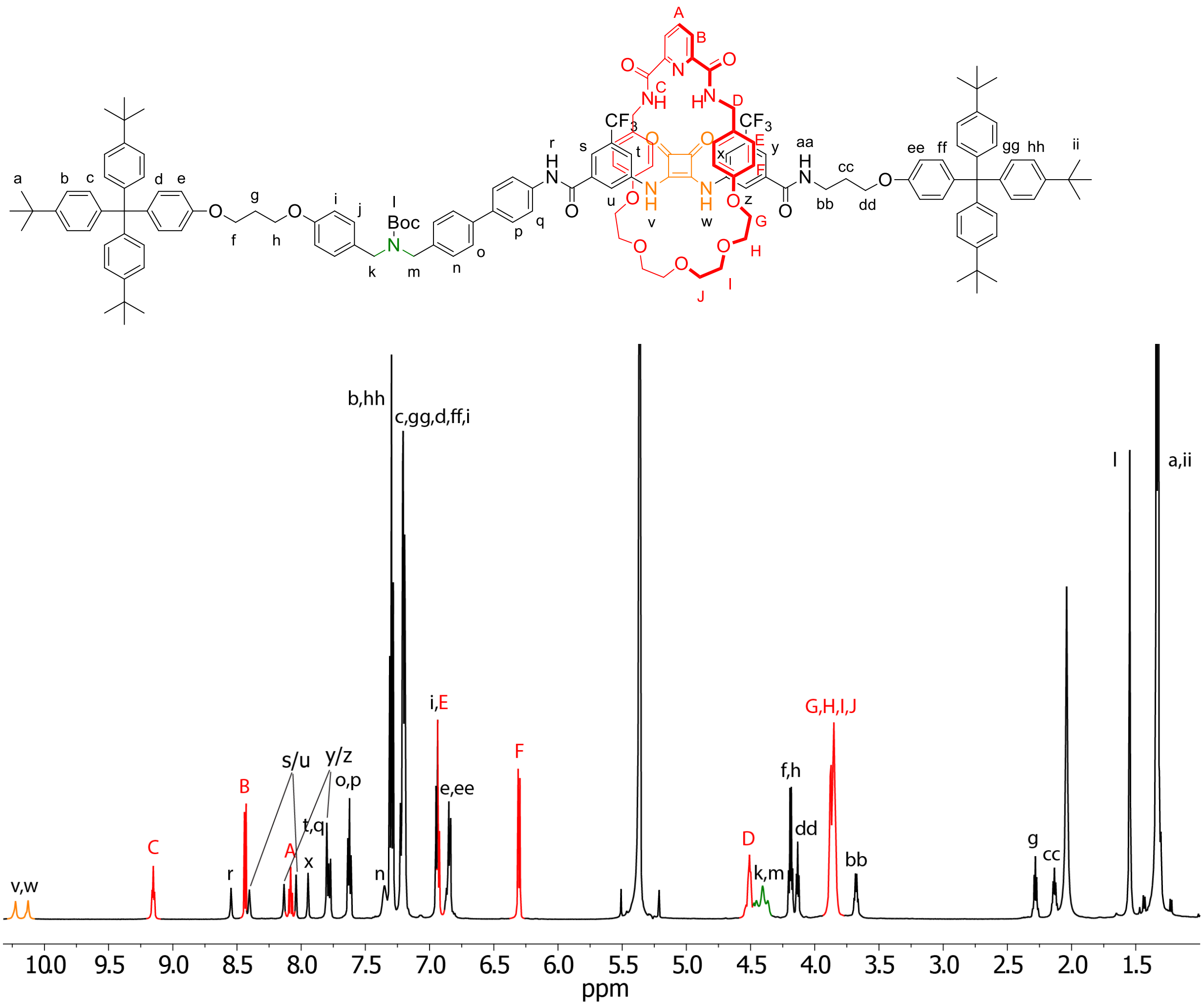

Figure S3. ^{1}H NMR spectrum (600 MHz, CD_2Cl_2) of compound **1**-Boc.

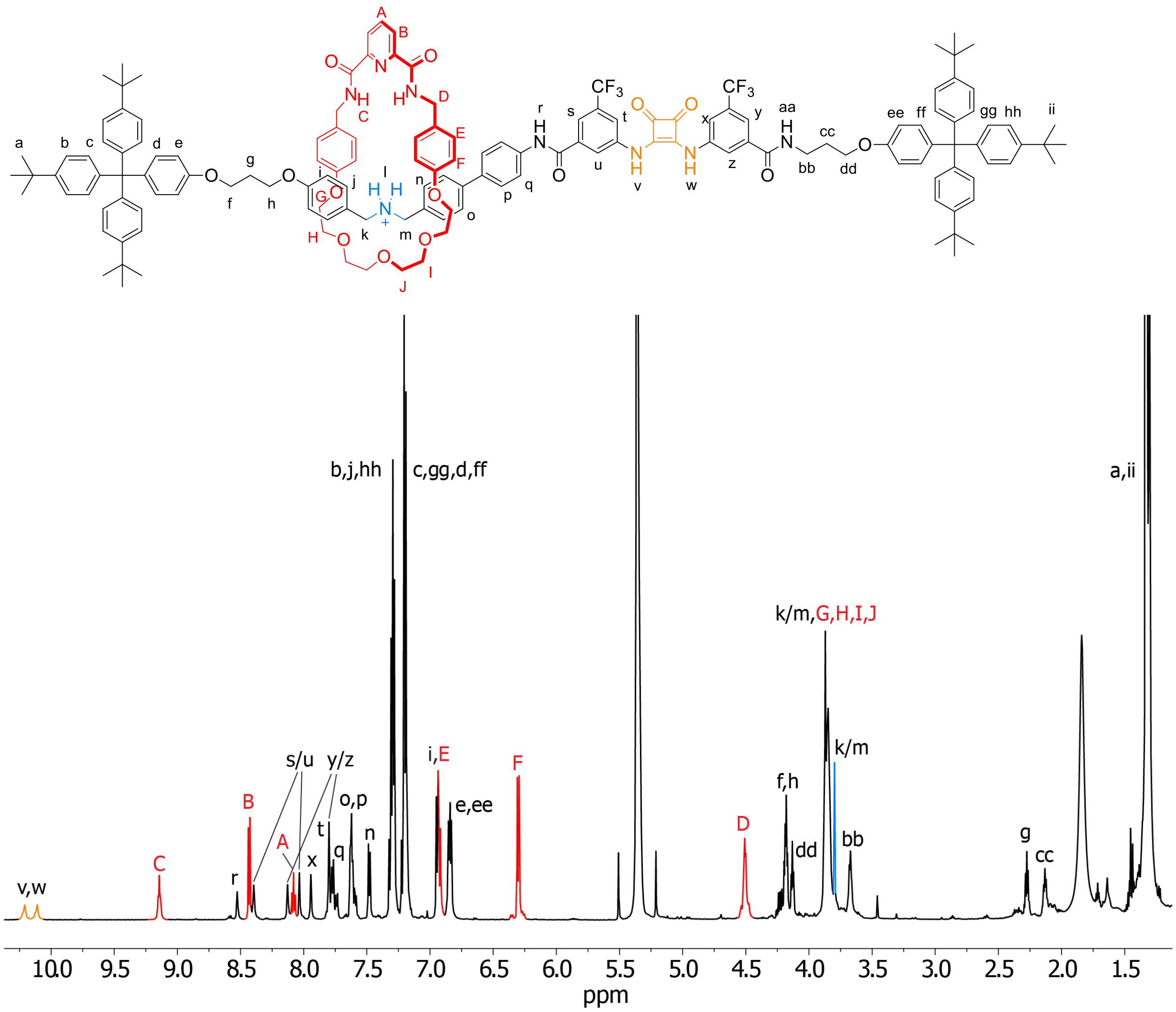

Figure S4. ^{1}H NMR spectrum (600 MHz, CD_2Cl_2) of compound **1**-$H^+ \cdot CF_3CO_2^-$.

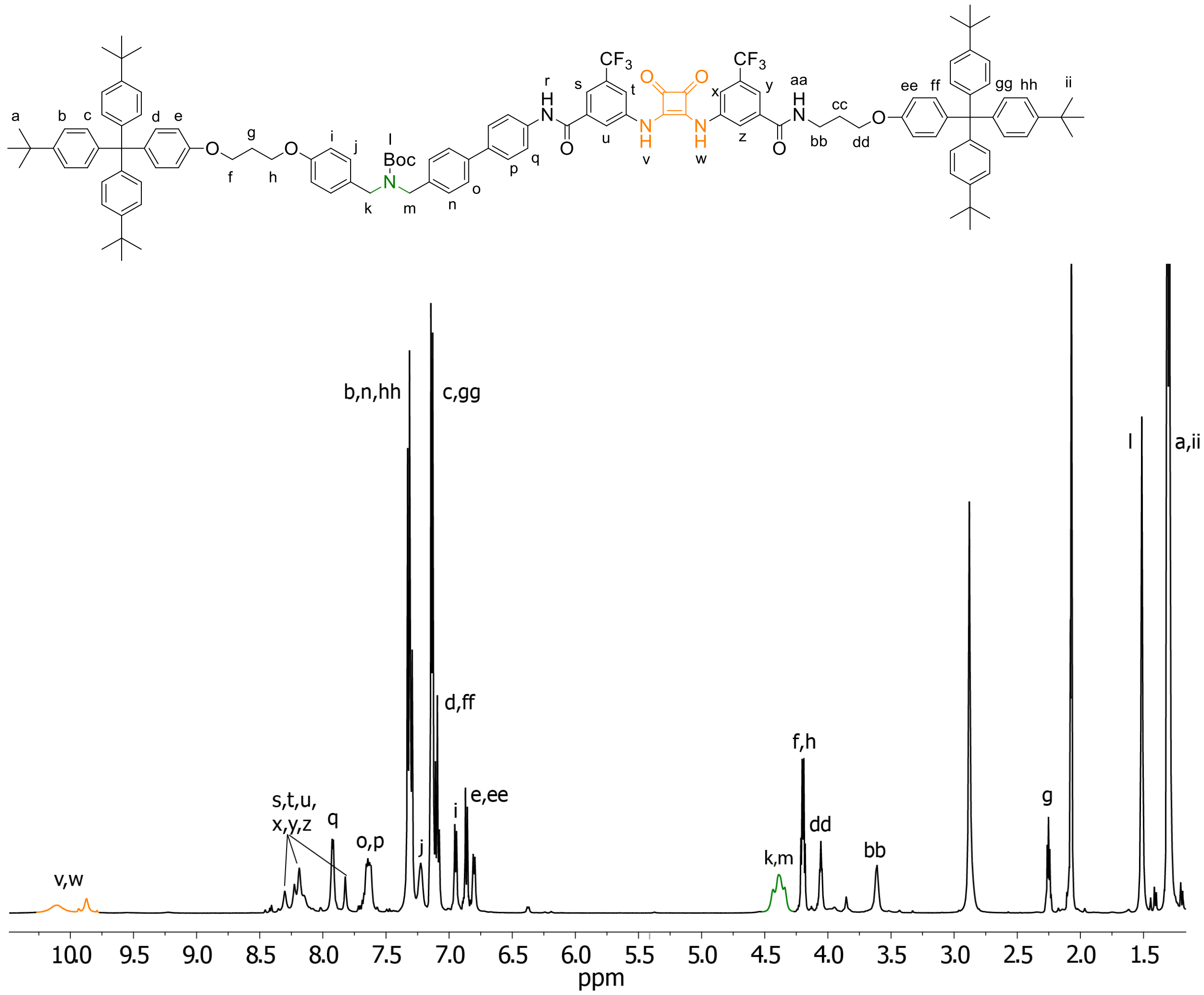

Figure S5. ^{1}H NMR spectrum (600 MHz, CD_3COCD_3) of compound **2**-Boc.

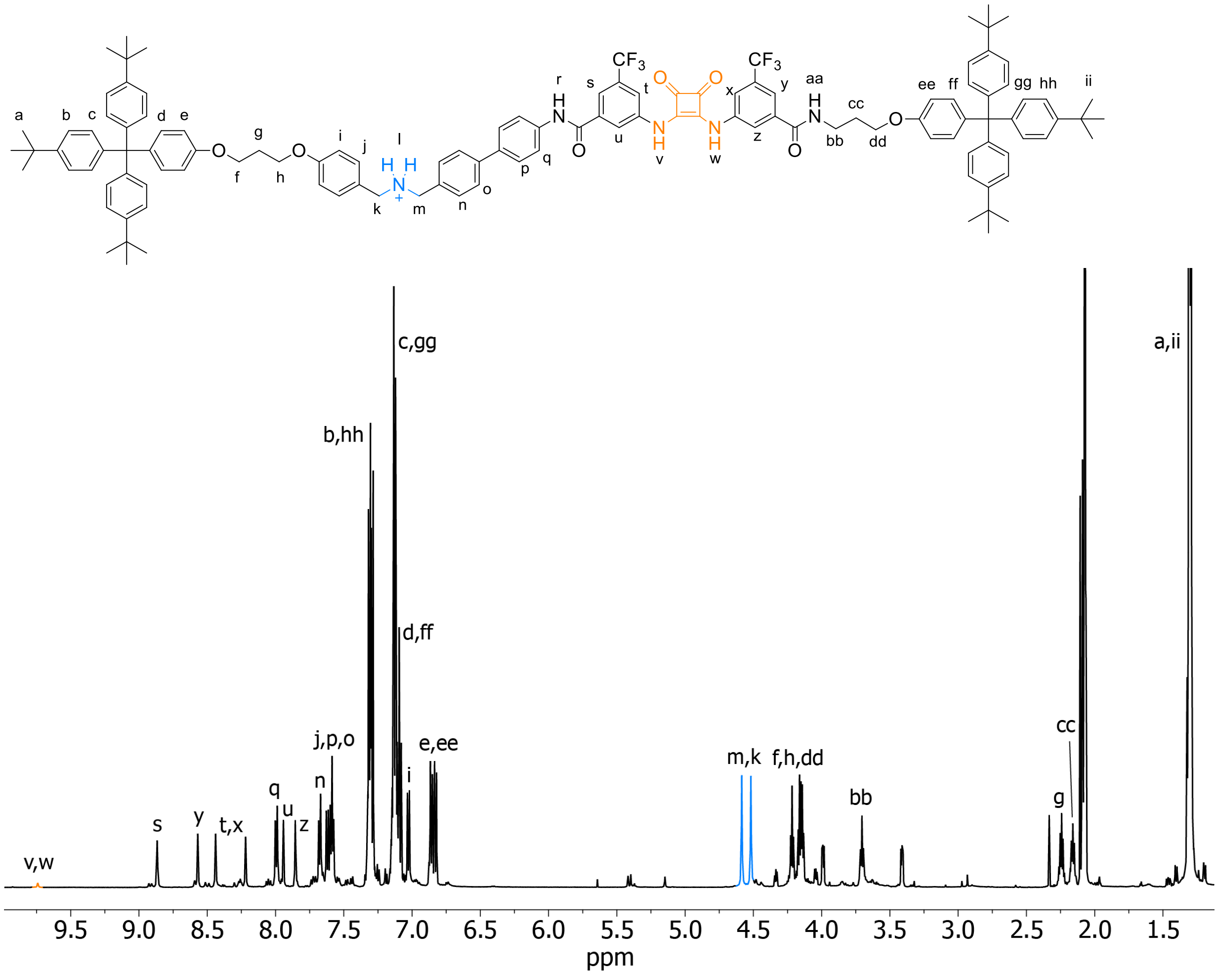

Figure S6. ^{1}H NMR spectrum (600 MHz, CD_3COCD_3) of compound **2**-$H^+\cdot CF_3CO_2^-$

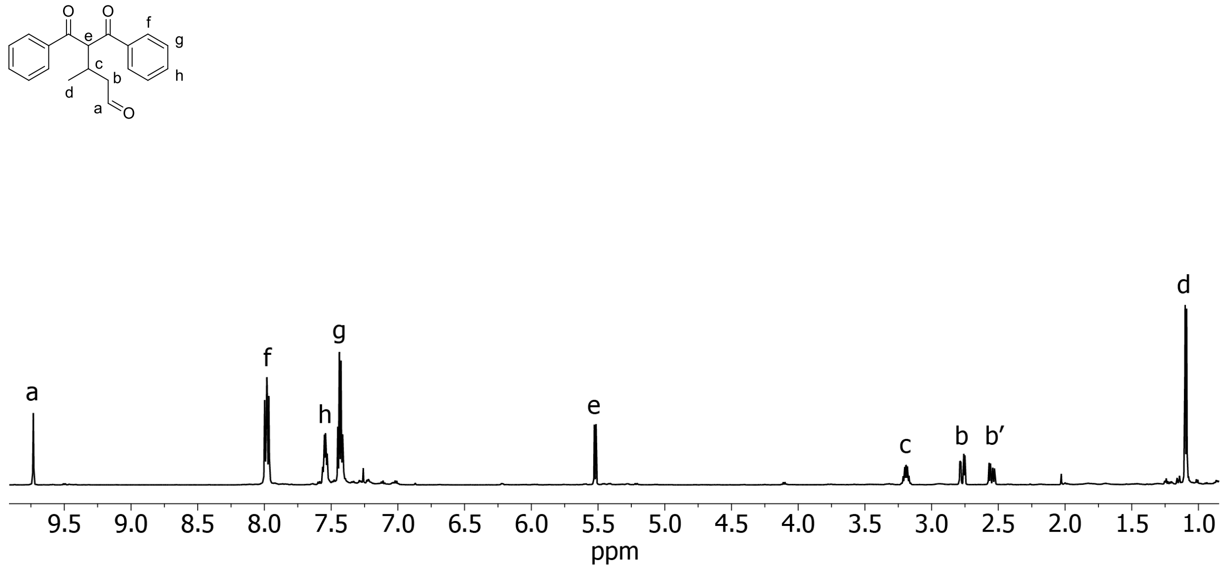

Figure S7. ^{1}H NMR spectrum (600 MHz, $CDCl_3$) of compound **10**.

2. Additional Supporting Figures

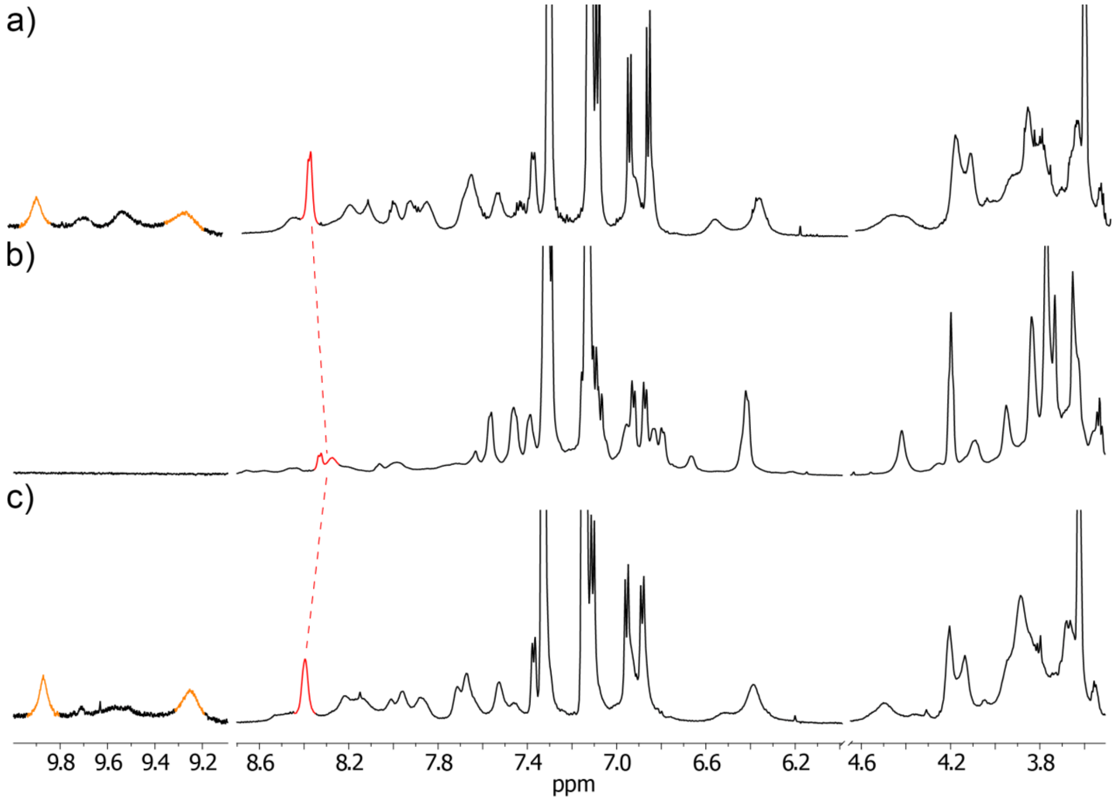

Figure S8. Partial ^1H NMR spectrum (600 MHz, CD_3COCD_3) of the reversible switching between a) **1**-$H^+ \cdot CF_3CO_2^-$, b) **1**, c) **1**-$H^+ \cdot CF_3CO_2^-$, using 2M $NaOH_{(aq)}$ (from a to b) and CF_3CO_2H (from b to c). The region between δ = 9.2 ppm and δ = 9.9 ppm is shown at x8 increased intensity in comparison with the signals below δ = 8.6 ppm

3. References and notes

1 H. W. Gibson, S. H. Lee, P. T. Engen, P. Lecavalier, J. Sze, Y. X. Shen, M. Bheda, *J. Org. Chem.* 1993, **58**, 3748.

2 J. D. Crowley, K. D. Hänni, A.-L. Lee, D. A. Leigh, *J. Am. Chem. Soc.* 2007, **129**, 12092.

3 M. J. Barrell, D. A. Leigh, P. J. Lusby, A. M. Z. Slawin, *Angew. Chem. Int. Ed.* 2008, **47**, 8036.

4 V. Blanco, D. A. Leigh, V. Marcos, J. A. Morales-Serna, A. L. Nussbaumer, *J. Am. Chem. Soc*. 2014, **136**, 4905; V. Blanco, D. A. Leigh, U. Lewandowska, B. Lewandowski, V. Marcos, *J. Am. Chem. Soc.* 2014, **136**, 15775.

5 The large number of ^{13}C signals observed is due to the different rotamers that arise from the slow rotation on the NMR timescale of the Boc protecting group.

6 Signal located under one of the residual peaks of the solvent and identified in the HSQC spectrum.

7 H. Zheng, W. Zhou, J. Lv, X. Yin, Y. Li, H. Liu, Y. Li, *Chem.—Eur. J.* 2009, **15**, 13253.

8 P. Kotrusz, S. Toma, H.-G. Schmalz, A. Adler, *Eur. J. Org. Chem.* 2004, 1577.
